# A Phyletically Rare Gene Promotes the Niche-specific Fitness of an *E. coli* Pathogen during Bacteremia

**DOI:** 10.1371/journal.ppat.1003175

**Published:** 2013-02-14

**Authors:** Travis J. Wiles, J. Paul Norton, Sara N. Smith, Adam J. Lewis, Harry L. T. Mobley, Sherwood R. Casjens, Matthew A. Mulvey

**Affiliations:** 1 Division of Microbiology and Immunology, Pathology Department, University of Utah School of Medicine, Salt Lake City, Utah, United States of America; 2 Department of Microbiology and Immunology, University of Michigan Medical School, Ann Arbor, Michigan, United States of America; National Institute of Allergy and Infectious Diseases, National Institutes of Health, United States of America

## Abstract

In bacteria, laterally acquired genes are often concentrated within chromosomal regions known as genomic islands. Using a recently developed zebrafish infection model, we set out to identify unique factors encoded within genomic islands that contribute to the fitness and virulence of a reference urosepsis isolate—extraintestinal pathogenic *Escherichia coli* strain CFT073. By screening a series of deletion mutants, we discovered a previously uncharacterized gene, *neaT*, that is conditionally required by the pathogen during systemic infections. *In vitro* assays indicate that *neaT* can limit bacterial interactions with host phagocytes and alter the aggregative properties of CFT073. The *neaT* gene is localized within an integrated P2-like bacteriophage in CFT073, but was rarely found within other proteobacterial genomes. Sequence-based analyses revealed that *neaT* homologues are present, but discordantly conserved, within a phyletically diverse set of bacterial species. In CFT073, *neaT* appears to be unameliorated, having an exceptionally A+T-rich composition along with a notably altered codon bias. These data suggest that *neaT* was recently brought into the proteobacterial pan-genome from an extra-phyletic source. Interestingly, even in G+C-poor genomes, as found within the Firmicutes lineage, *neaT*-like genes are often unameliorated. Sequence-level features of *neaT* homologues challenge the common supposition that the A+T-rich nature of many recently acquired genes reflects the nucleotide composition of their genomes of origin. In total, these findings highlight the complexity of the evolutionary forces that can affect the acquisition, utilization, and assimilation of rare genes that promote the niche-dependent fitness and virulence of a bacterial pathogen.

## Author Summary

Bacterial pathogens, even those belonging to the same species, can be incredibly diverse with regard to the genes they carry. However, the design of vaccines and antibiotics typically relies upon identification of general molecular features shared by the targeted organisms. Thus, we have traditionally focused on broadly conserved characteristics of pathogenic bacteria, often ignoring the genes that account for their individuality. In this article we report the discovery of a unique gene, *neaT*, that promotes the fitness of a pathogenic *Escherichia coli* isolate in zebrafish and mouse models of systemic blood infections. Surprisingly, *neaT* is rarely found in other related strains of *E. coli* and appears to have been recently acquired from distant lineages of bacteria via a process known as ‘lateral gene transfer’ that is used by microbes to swap genetic material. Expression of the *neaT* gene appears to help *pathogens* avoid interactions with host immune cells, possibly by altering bacterial surface structures. This work provides an interesting example of how the lateral acquisition of a rare gene can impact the niche-specific virulence properties of a pathogen, shedding light on the mechanisms that drive pathogen evolution and diversity.

## Introduction

As a species, *Escherichia coli* is best known for colonizing the lower intestine of humans and other warm-blooded vertebrates [1], [2]. The contingent exit from the intestinal tract presents strains of *E. coli* with a multitude of secondary habitats, including host-associated and free-living niches [2], [3], [4], [5], [6]. A subset of *E. coli* designated **ex**traintestinal **p**athogenic ***E*** *.* ***c*** *oli* (ExPEC) excels at colonizing host-associated extraintestinal environments, resulting in an array of human diseases including urinary tract infections, bacteremia, and meningitis [7]. ExPEC strains also exhibit an impressive zoonotic capacity, being able to persist and cause disease in a variety of domesticated animals [8], [9], [10], [11]. Collectively, ExPEC-related diseases represent daunting medical, agricultural, and economic burdens that threaten to worsen as antibiotic-resistant strains become more prevalent [8], [12], [13]. The evolutionary forces that underlie the emergence and niche tropisms of ExPEC have yet to be completely defined. Considering gene content, substantial intra-specific variation often exists between bacterial isolates, particularly among strains of pathogenic *E. coli*. Key questions regarding the origin of this heterogeneity and its impact on the fitness of virulent strains remain unanswered.

Bacteria are proficient at rapidly developing innovative, selectable traits to maintain fitness within complex environments—a property known as ‘evolvability’ [14], [15], [16], [17], [18]. Despite being largely asexual organisms that multiply by binary fission, bacteria engage in a genetically promiscuous behavior known as ‘lateral gene transfer’ (LGT). Laterally acquired genes can provide context-specific functions, such as the ability to metabolize atypical substrates [19], adhere to a variety of surfaces [7], neutralize antibiotics and other toxic compounds [5], or participate in niche construction [20]. Bacteria have several means of obtaining potentially beneficial elements through LGT: direct acquisition from the environment (transformation), transfer through cell-to-cell mating (conjugation), and acquisition from bacterial viruses known as bacteriophages (transduction) [14], [21], [22], [23], [24]. It has been estimated that ∼81% of all genes within a bacterial chromosome have been involved in LGT at some point, suggesting that this behavior is not just an anomalous event, but that over time it is a foundational component of bacterial evolution [25].

The genomes of *E. coli* are laden with the signatures of past LGT events. Since the first genome sequencing projects it has been apparent that *E. coli* chromosomes are highly mosaic [26], [27], [28]. In part, this chromosomal architecture results from the presence of ‘genomic islands’ (GI) that intermittently disrupt synteny [29], [30], [31], [32], [33], [34]. Many GIs exhibit clear signs of having been involved in past LGT events as they are often in proximity to mobile elements, such as transposons, or are themselves integrated phages or plasmids [35]. Accompanying this interchangeable chromosomal arrangement is a vast superset of genes defined as the pan-genome [32], [36], [37]. Whereas an average *E. coli* genome contains about 4,700 genes, the pan-genome of this species is estimated to be over 17,000 genes. Most *E. coli* strains share a subset of the pan-genome, which encodes vertically inherited genes that dictate the fundamental cellular properties of the lineage. This core genome surprisingly accounts for only 40–50% of the genetic makeup of any particular isolate. The rest of the chromosome contains strain-specific combinations of genes that are infused throughout the core genome and encode a variety of accessory functions that can provide unique selective advantages [32], [37], [38].

With this information in mind, we systematically screened GIs of a urosepsis ExPEC isolate for laterally acquired genes that affect virulence in a surrogate zebrafish host model. We identified a novel gene—designated *neaT* (**n**omadically **e**volved **a**cyl**t**ransferase)—that is required during blood-borne, but not localized, infections in both zebrafish and mice. The *neaT* locus was unexpectedly rare in the genomes of closely related *E. coli* strains and other Proteobacteria, suggesting that it was obtained from outside the contemporary *E. coli* pan-genome. Proteobacterial *neaT* homologues, in general, exhibit a high degree of allelic variance, have reduced guanine and cytosine (G+C) content, and are often localized within the integrated genomes of unrelated bacteriophages. These observations indicate that *neaT*-like alleles may have been recently acquired on multiple occasions by the proteobacterial supraspecies pan-genome. Together, our results provide molecular and bioinformatic evidence that the acquisition of unique genes like *neaT* during the evolution of particular ExPEC isolates can significantly impact bacterial fitness and virulence within specific host environments. Possible evolutionary forces that generate the observed sequence-level features of *neaT* and the role that bacterial individuality plays in pathogenesis are considered.

## Results

### The P2-like prophage b0847 promotes the fitness and virulence of ExPEC strain CFT073 during systemic infection of zebrafish embryos

The ExPEC strain CFT073 was isolated from the blood of a human patient with acute pyelonephritis (kidney infection) [26], [39]. This urosepsis isolate is versatile, with the apparent ability to traverse several host microenvironments to reach the bloodstream, and has a relatively large genome of 5,369 protein-coding genes and several GIs. In previous work, we found that CFT073 is exceptionally lethal in an infection model that uses zebrafish embryos as surrogate hosts for the high-throughput analysis of ExPEC virulence [40]. At 48 h post-fertilization (hpf), zebrafish possess an innate immune system composed primarily of phagocytic cells and antimicrobial peptides [41], [42], [43], [44]. These defenses mirror those employed by mammalian hosts to combat ExPEC.

**Figure 1:**
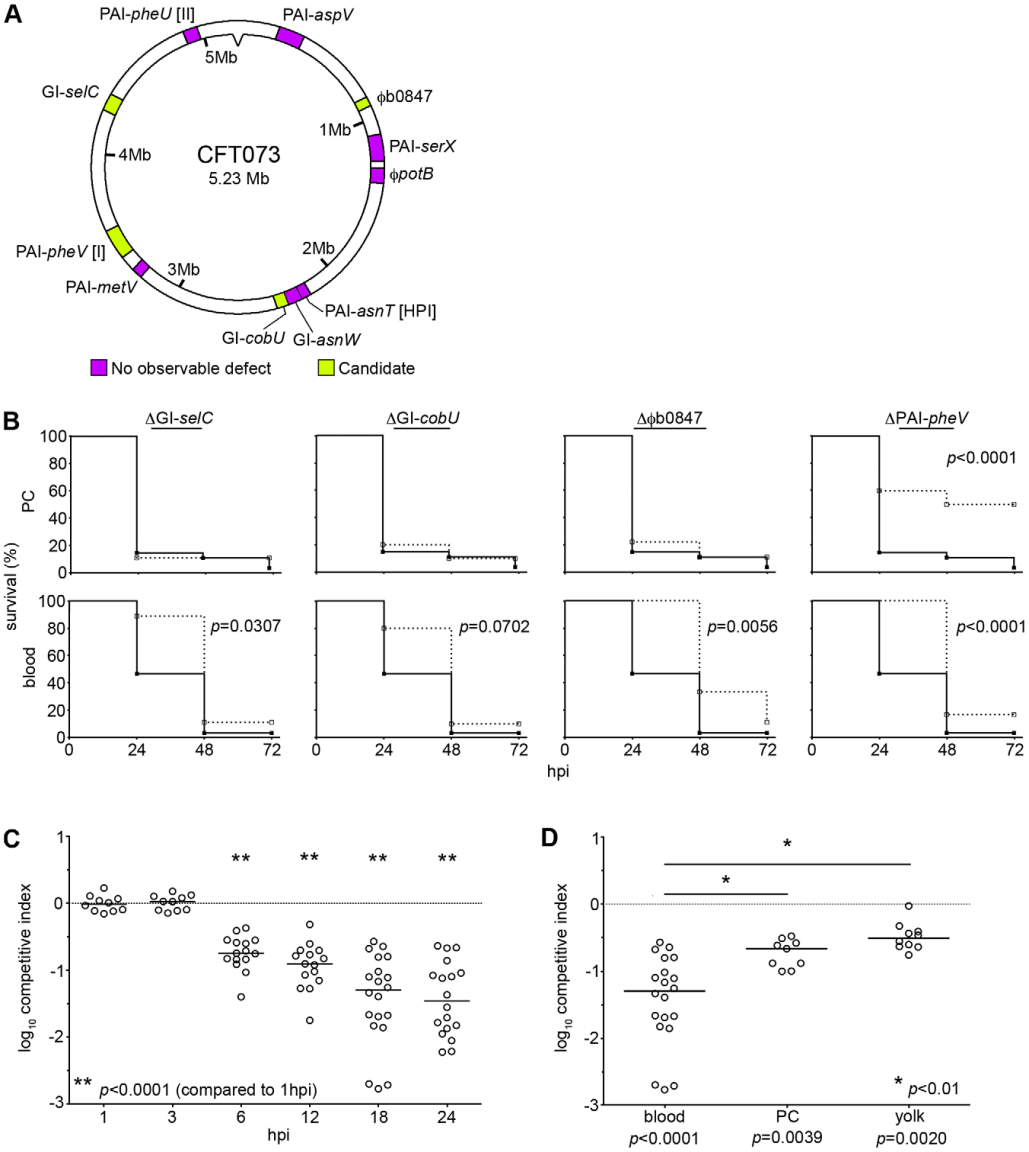
The φb0847 island is important for CFT073 pathogenicity during systemic infection in zebrafish embryos. (A) Diagram of GIs and their location within the CFT073 chromosome that were screened in the zebrafish host. Magenta indicates island mutants that had no observable defects, while green denotes island mutants that displayed significant attenuation. (B) The pericardial cavity (PC, top row) and blood (bottom row) of 48 hpf embryos were inoculated with 1,000–2,000 CFU. Fish were scored for death at 0, 24, 48, and 72 h post-inoculation (hpi). Data are presented as Kaplan-Meier survival plots and *p* values were calculated using a log-rank (Mantel-Cox) test (sample sizes for each curve are listed in Table 1). (C) Equal numbers (1,000–2,000 CFU total) of wild type and CFT073Δφb0847 were inoculated into the bloodstream of embryos. Fish were sacrificed and bacterial loads enumerated at the indicated times by differential plating (*n* = 10 to 20 embryos). Data are represented as competitive indices, where negative values indicate a reduction in fitness of the mutant strain. (D) Bacteria were prepared as in (C) and inoculated into the PC or yolk. Fish were sacrificed at 18 hpi and bacterial numbers determined (*n* = 9–10 embryos). Data from blood infections is the same as in (C), provided as a reference. *P* values were determined using two-tailed Mann-Whitney *t* tests. Median values are indicated by bars in (C) and (D).

To identify GI-associated virulence factors carried by CFT073, we screened 11 previously described deletion mutants that each lack a specific GI (Table 1 and Figure 1A) [45]. In blinded assays, 48 hpf zebrafish embryos were infected with 1,000 to 2,000 colony-forming units (CFU) of either wild type CFT073 or one of the 11 GI mutants. Bacteria were delivered into one of two injection sites: the fluid-filled sac surrounding the heart referred to as the pericardial cavity (PC), which mimics a localized tissue infection, and the circulation valley (CV), which facilitates rapid dispersal of bacteria into the bloodstream [40]. Each of these sites likely challenges the pathogen with different nutrient limitations, receptor availability, and host defenses.

**Table 1:** Summary of GI screen in zebrafish infection sites.

GI deletion variant	Size (Kbp)	PC		Blood	
		*n*	*p* value	*n*	*p* value
Φ*potB*	44	9	0.2272	10	0.3581
GI-*selC*	68	9	0.6862	9	**0.0307**
GI-*cobU*	44	10	0.6689	10	0.0702
φb0847	33	9	0.2831	9	**0.0056**
GI-*asnW*	54	10	0.1338	10	0.9703
PAI-*asnT* [HPI]	32	10	0.7036	10	0.6317
PAI-*pheV* [I]	123	30	**<0.0001**	30	**<0.0001**
PAI-*metV*	32	10	0.7586	9	0.179
PAI-*pheU* [II]	52	10	0.2322	10	0.6321
PAI-*aspV*	100	10	0.2322	10	0.7972
PAI-*serX*	113	9	0.4113	10	0.6163

*n* denotes number of individual embryos used.

*p* values reflect the statistical significance of differences between wild type and mutant killing kinetics.

In this infection model, increased growth of ExPEC is associated with decreased survival of the host [40]. All 11 GI mutants, with the exception of ΔGI-*aspV*, grew equally well in broth culture at 28.5°C and 37°C (data not shown). Following inoculation into the PC, only deletion of the 123 kb GI PAI-*pheV* [I] resulted in a significant decrease in virulence relative to wild type CFT073 (Figure 1B, top). This was not surprising as PAI-*pheV* [I] encodes the notable ExPEC-associated virulence factors α-hemolysin (pore-forming toxin), SAT (vacuolating toxin), P pili (adhesive organelles), aerobactin (iron acquisition system), and K2 capsule (immune evasion). The ability of the ΔPAI-*pheV* mutant to still kill approximately half of the embryos suggests that additional factors with overlapping roles in virulence within the PC are encoded outside of PAI-*pheV* and the 10 other GIs tested.

The ΔPAI-*pheV* mutant was also attenuated following inoculation of the CV to initiate systemic infection, as were the GI mutants ΔGI-*selC*, ΔGI-*cobU*, and Δφb0847 (Figure 1B, bottom). In addition to several hypothetical genes, the *selC* and *cobU* islands harbor genes that appear to be components of polyamine and iron transport systems, respectively. Both polyamines and iron acquisition systems are known to be important mediators of ExPEC fitness in mouse models of infection [46], [47], [48]. Although the ΔGI-*cobU* mutant exhibited only a modest reduction in virulence in these assays using inoculation doses of 1,000–2,000 CFU/embryo, with slightly higher doses between 2,000 to 3,000 CFU/embryo this mutant displayed more dramatic and significant (*p*<0.05) attenuation (Table S1). This observation supports previous findings indicating that the inoculation dose can markedly influence the discernibility of some mutant phenotypes in the zebrafish host [40].

**Figure 2:**
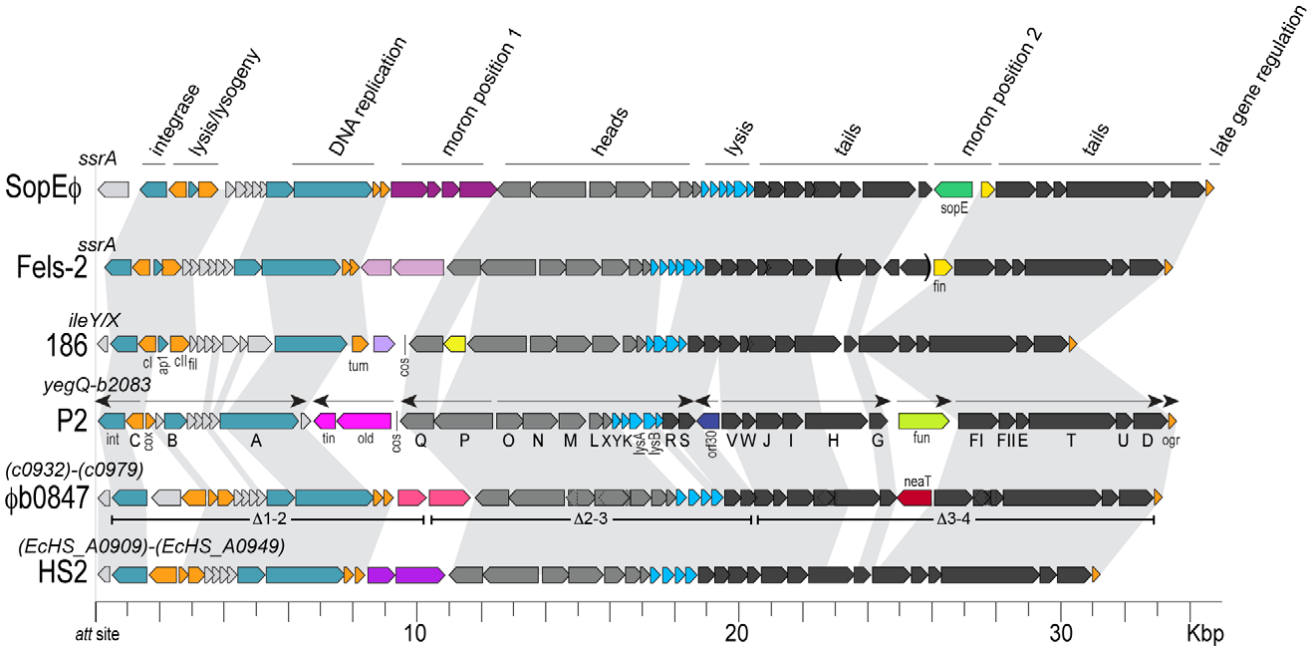
Alignment of φb0847 genome to other P2-like bacteriophage. Related P2-like prophages are aligned relative to their respective integration sites (att). Size is measured in kilobase pairs (Kbp). E. coli phages P2 and 186 and Salmonella phages Fels-2 and SopE5 were previously characterized. HS2 is an uncharacterized prophage contained within the genome of the commensal E. coli strain HS. Our unpublished analysis indicates that P2, 186, and Fels-2 represent three different “sequence types” based on virion proteins, which are typically >85% identical within each of these three groups and 50–70% identical between the groups. Bracketed numbers below φb0847 indicate positions of the deletion mutants generated to assess the functionality of genes within broad regions of φb0847. The neaT gene is distinguished by a red open reading frame in moron position 2.

The remaining GI showing a phenotype in our screen is composed of an intact integrated phage genome (prophage) named ‘φb0847’ (Figure 1B) [45]. This prophage is 33 kb in length and contains 48 predicted open reading frames, most of which encode recognizable phage proteins that share homology with genes of tailed phages belonging to the order *Caudovirales*. More specifically, φb0847 carries genes involved in regulation, replication, and virion assembly that are related to and syntenic with the genes of phage P2 and its relatives (Figure 2). From this analysis, it is clear that the φb0847 prophage is a member of the P2-like phage group and likely represents a fully functional phage genome complete with all the essential genes associated with P2-like phages [49]. Aside from the Δ*pheV* mutant, with its fairly well characterized assortment of virulence genes, Δφb0847 displayed the most pronounced defect of the island mutants examined. Therefore, the φb0847 GI became the primary focus of our investigation.

To further define the contribution of φb0847 to the virulence and fitness of CFT073, we carried out competitive assays in which a one-to-one mixture of wild type and mutant bacteria were injected into the CV of zebrafish embryos (Figure 1C). At the indicated time points, the infected embryos were homogenized and bacteria present were enumerated by dilution plating on selective agar. Δφb0847 carries a kanamycin resistance cassette that was used to distinguish wild type and mutant strains. No differences between wild type CFT073 and the Δφb0847 mutant were observed until 6 h post-inoculation (hpi), when Δφb0847 titers began to decline (Figure 1C). These results indicate that the φb0847 island is dispensable during initial stages of a systemic infection, but enhances bacterial fitness at later time points, coordinate with the upregulation of host inflammatory responses engage. The Δφb0847 mutant displayed more modest, though still significant, decreases in fitness during competitive assays against wild type CFT073 within the PC and yolk sac at 18 hpi (Figure 1D). Phagocytes are recruited into the PC *en masse* in response to infection with ExPEC [40], possibly contributing to the competitive disadvantage of the Δφb0847 mutant within this niche. On the other hand, the yolk is a rich source of nutrients for bacteria and is mostly free of phagocytes and other immunosurveillance mechanisms. However, the yolk does contain maternally inherited antimicrobial compounds that could account for the slight reduction in fitness of Δφb0847 within this host environment [50]. Competitive experiments in broth culture did not reveal appreciable differences between the wild type and mutant strains (data not shown).

### φb0847 harbors a multigenic region that contributes to fitness

**Figure 3:**
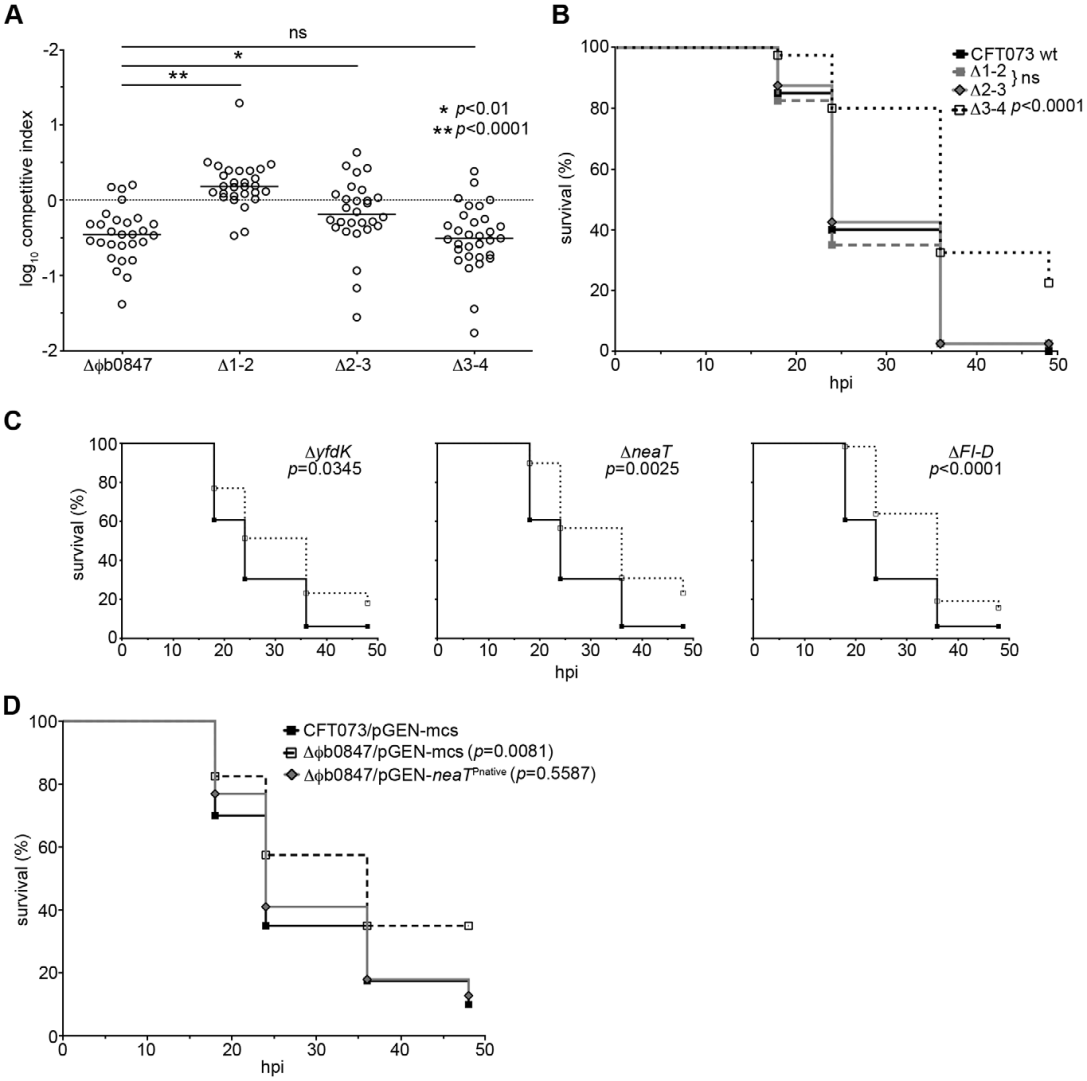
φb0847 harbors multiple loci that contribute to the fitness of CFT073 during systemic challenge. (A) Equal numbers (1,000–2,000 CFU total) of wild type CFT073 and each mutant derivative indicated were inoculated into the bloodstream of embryos. Fish were sacrificed and bacterial loads enumerated at 12 hours post inoculation (hpi) by differential plating (*n*>28). Data are presented as competitive indices with negative values indicating a reduction in fitness of the mutant. *P* values were determined using two-tailed Mann-Whitney *t* tests; bars indicate median values. (B), (C), and (D) 1,000–2,000 CFU of wild type CFT073, the indicated mutant, or recombinant derivative were each inoculated into the blood of 48 hpf embryos. Fish were scored for death every 6 h starting at 18 hpi until 48 hpi (*n* = 40 or more embryos for each curve). pGEN-mcs in (D) serves as an empty vector control for pGEN-*neaT* ^Pnative^. Data in (B), (C), and (D) are presented as Kaplan-Meier survival plots. A log-rank (Mantel-Cox) test was used to determine *p* values; ns = not significant.

To identify genes within φb0847 that, when deleted, recapitulate the attenuated phenotypes of Δφb0847, we constructed partial deletion mutants lacking one of three nearly equal-sized regions of the prophage island (designated Δ1–2, Δ2–3, and Δ3–4, as indicated along the φb0847 genome in Figure 2). In competitive assays, the Δ1–2 and Δ2–3 mutants were significantly more fit than the full Δφb0847 mutant at 12 hpi (Figure 3A). Analysis at 12 hpi allowed time for selection to take place, while limiting artifacts due to bacterial replication at later time points in dead and dying hosts where selective pressures are presumably weaker. In these assays, only the Δ3–4 mutant phenocopied the complete φb0847 island deletion mutant (Figure 3A). Lethality of this mutant variant was also significantly reduced in comparison to wild type CFT073 and the Δ1–2 and Δ2–3 mutants in independent challenges (Figure 3B). These results indicate that one or more genes within the terminal 3–4 region of the φb0847 prophage enhances both the fitness and virulence of CFT073 during systemic infections within the zebrafish host.

Temperate prophage genomes like φb0847 can carry ‘lysogenic conversion’ genes that affect the bacterial host but are not essential for lytic phage growth. To avoid disruption of critical phage processes, the integration of this genetic material is generally tolerated only in certain regions of the prophage genome. These added sequences are known as ‘morons’, because bacteriophages with such insertions have *more* DNA [51], [52]. Moron genes typically contain their own regulatory elements and vary among individual phage genomes. They often alter the surface structure or physiology of the bacterial host and can benefit the phage by making its host refractory to competing parasites or otherwise promoting bacterial survival and growth [52], [53].

The P2-like phages appear to have at least two variable moron positions (Figure 2). Using phage P2 as a reference, the location of moron position 1 is between the DNA replication gene *A* and head assembly gene *Q*, and moron position 2 is between the tail fiber gene *G* and tail sheath gene *FI* (Figure 2) [49]. In φb0847 within CFT073, the second moron site, which is absent from the Δ3–4 mutant, contains one open reading frame that is oriented in the opposite transcriptional direction to the flanking tail genes. This gene, which we named *neaT* for reasons described later, encodes a putative acyltransferase (Pfam:PF01757). This gene is not conserved among P2-like phages and is likely not critical for lytic replication of φb0847.

In light of this information, *neaT*, the immediately proximal gene *yfdK* (homologous to P2 phage tail gene *G*), and the collection of distal tail genes (*FI* through *D*) were individually deleted from the φb0847 prophage in CFT073. All three mutant derivatives—Δ*yfdK*, Δ*neaT*, and Δ*FI-D*—were attenuated in their ability to kill zebrafish embryos after injection into the blood via the CV (Figure 3C). Despite the significantly reduced virulence of these mutants, no defects in fitness were observed in competitive assays with wild type CFT073 (data not shown). The lack of any discernable fitness defects in competition assays may 1) reflect the ability of wild type CFT073 to trans-complement the mutant strains *in vivo* and/or 2) indicate that there is cooperative interplay among the *yfdK*, *neaT*, and *FI-D* loci. Of note, disruption of loci flanking *neaT* did not appreciably alter its expression in broth culture (Figure S1). Furthermore, we found no evidence that the *neaT* mutant could be complemented *in vivo* during competition assays by acquiring φb0847 sequences from the wild type strain (Figure S2). Interestingly, a *yfdK* homologue was recently shown to aid the survival of a K-12 laboratory strain of *E. coli* in acidic environments [54], but no mechanism for this effect is known, and to the authors' knowledge, *yfdK* homologues have not been implicated in pathogenesis.

### The *neaT* gene restores virulence to the φb0847 island deletion mutant

The *in vivo* assays presented in Figure 3C and bioinformatic analyses described below highlight *neaT* as a gene of potential importance to the fitness and virulence of CFT073. To test this possibility, the *neaT* locus, including an upstream promoter region of 211 bp, was amplified from the CFT073 chromosome and cloned into the high-retention plasmid pGEN-mcs, yielding pGEN-*neaT* ^Pnative^. Semi-quantitative reverse transcription polymerase chain reaction (RT-PCR) indicated that *neaT* transcript levels made from the pGEN-*neaT* ^Pnative^ vector in broth culture were about 1.7-fold higher than those observed in wild type CFT073 (Figure S3). Complementation experiments were performed comparing the lethality of wild type CFT073/pGEN-mcs, Δφb0847/pGEN-mcs, and Δφb0847/pGEN-*neaT* ^Pnative^ in zebrafish embryos after inoculation of the CV (Figure 3D). The complete prophage deletion mutant Δφb0847 carrying the empty vector pGEN-mcs exhibited a significant delay in killing relative to either the wild type strain CFT073/pGEN-mcs or the complemented mutant Δφb0847/pGEN-*neaT* ^Pnative^. In total, these experiments identify *neaT* as a virulence determinant contained within the φb0847 island of CFT073; therefore, the uncharacterized *neaT* gene became the focal point for the remainder of our investigation.

### *neaT* is required for fitness during systemic, but not localized infections in a mammalian host

**Figure 4:**
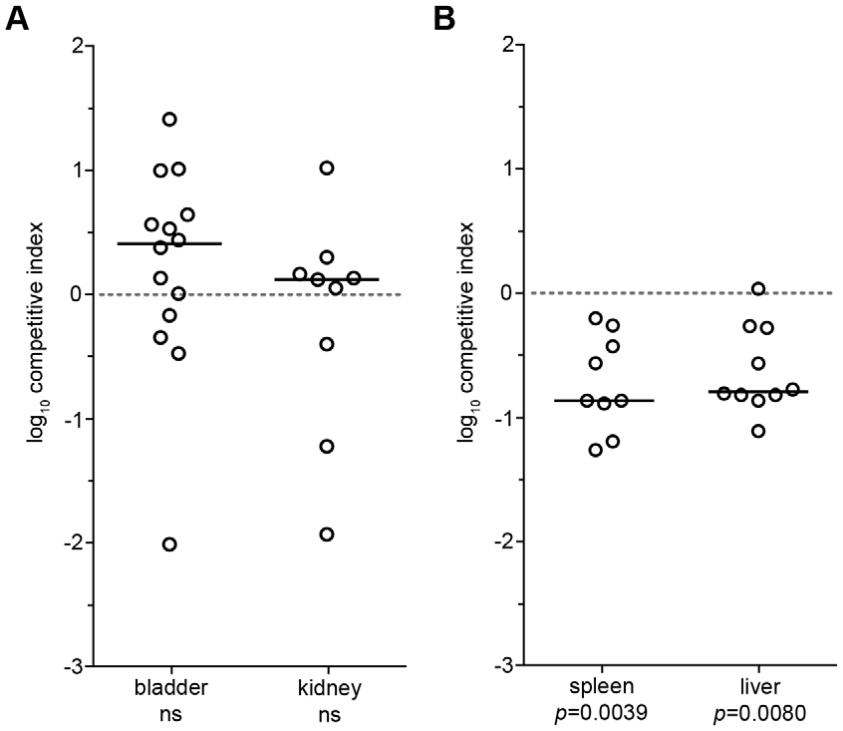
*neaT* enhances the fitness of CFT073 in a murine model of bacteremia. (A) Equal numbers (10^7^ CFU total) of wild type CFT073 and CFT073***Δ*** *neaT* were transurethrally inoculated into the bladder of CBA/J female mice. Mice were sacrificed, organs harvested, and bacterial loads enumerated at 3 d post inoculation. (B) Equal numbers (10^6^ CFU total) of wild type and CFT073Δ*neaT* were inoculated into the bloodstream of CBA/J female mice via tail vein injection and bacterial titers present in the spleen and liver were enumerated 24 h later. Data are shown as competitive indices, where negative values indicate a reduction in the fitness of CFT073Δ*neaT*. Bars indicate median values for each group; *n*≥9 mice. *P* values determined using Wilcoxon-matched paired signed rank; ns = not significant.

To extend our observations made using zebrafish, we employed a murine model to further define the requirement for *neaT* during localized and systemic infections. For localized challenges, we took advantage of a well-characterized mouse model of urinary tract infection. Wild type CFT073 and the Δ*neaT* mutant were mixed at a one-to-one ratio and inoculated via transurethral catheterization into adult female CBA/J mice. After 3 days, animals were sacrificed and bacterial titers within the bladders and kidneys were enumerated, revealing no outright competitive advantage for wild type CFT073 over the Δ*neaT* mutant in either organ (Figure 4A). To appraise the requirement for *neaT* during systemic infections, we utilized a recently described sub-lethal bacteremia model in which CBA/J mice were injected with a one-to-one mixture of wild type and mutant bacteria via the tail vein [55]. At 24 hpi the Δ*neaT* mutant was recovered at significantly reduced levels from the spleen and liver compared to wild type CFT073 (Figure 4B). These results confirm and extend our findings in the zebrafish host, demonstrating that *neaT* provides niche-specific advantages to CFT073 during systemic infections.

### Diversity and phage association of NeaT homologues

There are no closely related homologues of NeaT in *E. coli*. Only four matches were found in the current NCBI collection of 170 RefSeq *E. coli* genomes (as of June 2012) that produce an alignment E value<10^−6^ with similarity over >50% of the NeaT protein length. A PCR-based survey for the presence of *neaT* within various clinical *E. coli* isolates corroborated our *in silico* observation that *neaT* is rare among this taxon (Figure S4). Out of 21 randomly chosen isolates, none carried the CFT073 *neaT* allele. Homologues of *neaT* are also rarely detected in P2-like phage genomes; among 45 randomly chosen P2-like phages and prophages in *E. coli*, *Salmonella*, *Shigella*, and *Enterobacter* that we examined, only φb0847 carries a *neaT*-like gene. The closest match to NeaT in the NCBI database is encoded by locus *Ent638_2581* of *Enterobacter sp.* 638, whose protein product is only about 33% identical to NeaT. We note that several more distantly related *neaT* homologues are present in the genomes of other temperate phages and prophages of the bacterial family *Enterobacteriaceae* (Table 2). They are found, for example, in the *Shigella flexneri* phage Sf6 genome and several uncharacterized prophages of *S. flexneri*, in *E. coli* phage φV10 and a nearly identical prophage in the Shiga toxin-producing *E. coli* isolate DEC4D, and in a putative prophage within *Citrobacter rodentium* strain ICC168. The above *Enterobacter* homologue *Ent638_2581* is also carried within a putative prophage that is similar to *Shigella* phage SfV. Each of these phage-associated *neaT* homologues is un-ameliorated with respect to its bacterial host genome (see below), and each lies within a known moron position in its phage genome. Because *neaT* homologues differ substantially in sequence conservation and are found in a variety of tailed-phages, *neaT*-like genes may have been laterally acquired by *Enterobacteriaceae* lineages on several occasions, possibly via phage. Multiple *neaT* acquisition events would indicate that this gene has an underlying evolutionary importance to either the phages themselves or their hosts. In considering its putative function (see Figures S5, S6, and Text S1), its apparent lateral acquisition, and its allelic variance within the proteobacterial lineage, this gene was named ‘*neaT*’—**n**omadically **e**volved **a**cyl**t**ransferase. In the following sections we explore the evolutionary history of this gene by analyzing the diversity and distribution of *neaT*-like genes in more detail.

**Table 2:** *neaT* homologues associated with bacteriophages.

			%GC		
Bacterial host strain	Associated phage genome	Gene ID	*neaT* homologue	bacterial genome	% identity to *neaT* ^CFT073^ (% query coverage)
*E. coli* CFT073	P2-like φb0847 prophage	26107260	30	51	100 (100)
*E. coli* DEC4D	ε15-like prophage	377941589	31	50	22 (96)
*E. coli* O157:H7	ε15-like phage φV10	89152472	31	49	22 (94)
*Enterobacter* sp. 638	unnamed lambdoid prophage	146312226	38	53	34 (98)
*C. rodentium* ICC168	unknown prophage fragment	283784796	37	55	32 (41)
*S. flexneri* serotype X	Sf6	33334172	42	51	29 (35)
*S. flexneri* VA-6	Sf6-like defective prophage	333006890	42	51	29 (35)
*S. flexneri* K-218	unknown prophage	333006144	39	51	34 (86)
*S. flexneri* 4343-70	unknown prophage	332759112	39	51	34 (86)

### Phyletic distribution of *neaT*

**Figure 5:**
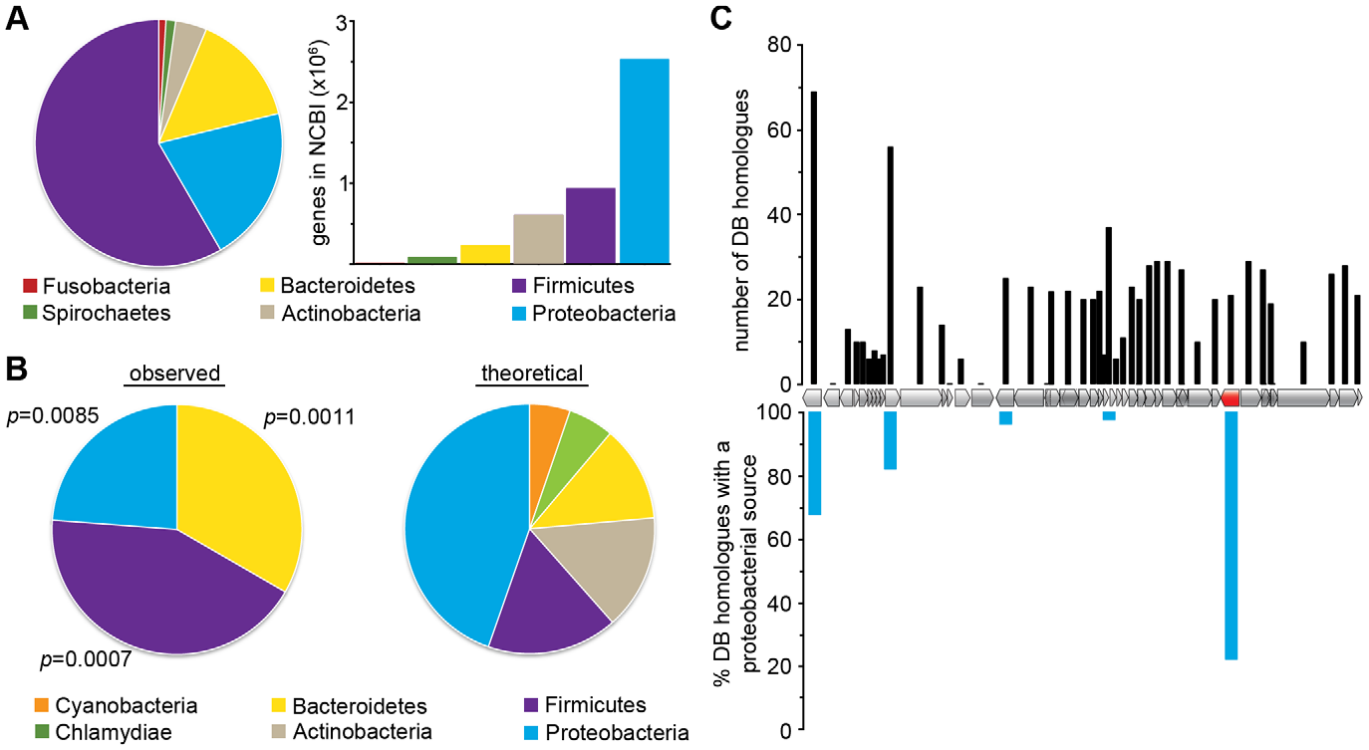
*neaT* is discordantly conserved. (A) Left: Phyletic distribution of neaT homologues among genomes retrieved from NCBI (n = 317). Right: Number of gene sequences deposited in NCBI for each phylum as of November 2011. (B) Left: Pie graph showing distribution of neaT homologues among each phylum represented in the custom database (n = 21 non-paralogous neaT genes). Right: Theoretical distribution of neaT homologues within phyla present in the custom database based on random chance. P values for the observed versus theoretical phyletic abundance of neaT homologues were calculated by .score analysis. (C) Upper y-axis: bar graph depicts total number of non-paralogous homologues retrieved from the custom database (DB) for each gene encoded within φb0847 (plotted along the x-axis with respect to its position within the prophage genome). The neaT open reading frame is indicated in red. Lower y-axis: bar graph showing the percent of proteobacterial homologuesfound in the homologue set for each φb0847 gene. Sequences unique to CFT073 were assigned 100% proteobacterial conservation.

To investigate the evolutionary source of *E. coli neaT* genes, we assessed the phyletic distribution of its homologues. BLASTp alignments were performed on the publically available NCBI database using NeaT from CFT073 as a probe for the search sets of Proteobacteria, Firmicutes, Bacteroidetes, Actinobacteria, Spirochaetes, and Fusobacteria [56]. Sequences were declared to be homologous if they had an alignment significance (E value) of <10^−6^ over >50% of their lengths [57]. These searches retrieved a total of 317 non-paralogous NeaT-like sequences. The distribution of phyla containing these sequences is depicted in Figure 5A (left), revealing that the majority of *neaT* homologues are from the Firmicutes and Bacteroidetes rather than Proteobacteria. In an attempt to control for the inherent bias of NCBI databases, we plotted the number of available gene sequences for each phylum represented in Figure 5A (right). This plot demonstrates that the high number of *neaT* homologues identified among non-proteobacterial phyla is not due to a skew in sequence abundances. To the contrary, total proteobacterial gene sequences overshadow those from other phyla and therefore underscore the relative rarity of *neaT* alleles in this taxon.

To quantify the phyletic distribution of NeaT homologues with greater statistical confidence, we performed bi-directional alignments of NeaT using BLASTp with a manually assembled database of open reading frames from a representative, yet broad, assortment of 165 phylogenetically classified bacterial genomes and associated plasmids obtained from NCBI (Table S3). This analysis confirmed that, compared to random chance, *neaT* homologues are significantly enriched among species belonging to the phyla Firmicutes and Bacteroidetes (Figure 5B). Moreover, many of the *neaT* homologues were detected in notable plant and animal pathogens, including *Erwinia spp.*, *Bacillus spp.*, *Staphylococcus aureus*, *Streptococcus oralis*, *Clostridium botulinum*, and *Porphyromonas spp*.

Results from similar alignments of *neaT* and all other φb0847-encoded genes are presented graphically in Figure 5C. For each prophage gene, the number of non-paralogous matches found in the custom database are represented as bars (upper axis) and the percent of those hits that are harbored within proteobacterial genomes (lower axis) are plotted against the position of the gene within φb0847 (x-axis). Given the host range of known P2-like phages, it is not unexpected that the majority of genes within φb0847 were exclusive to the proteobacterial phylum. Exceptions, in addition to *neaT*, include homologues of φb0847 genes encoding the phage integrase and a Dam methylase. However, *neaT* is unique among the φb0847 prophage genes in that over 75% of its matches (16 of 21) were from outside the Proteobacteria (Figure 5C and Table S4). The discordant conservation of *neaT* highlights its likely extra-phyletic origin.

### *neaT* displays signatures of recent lateral transfer

**Figure 6:**
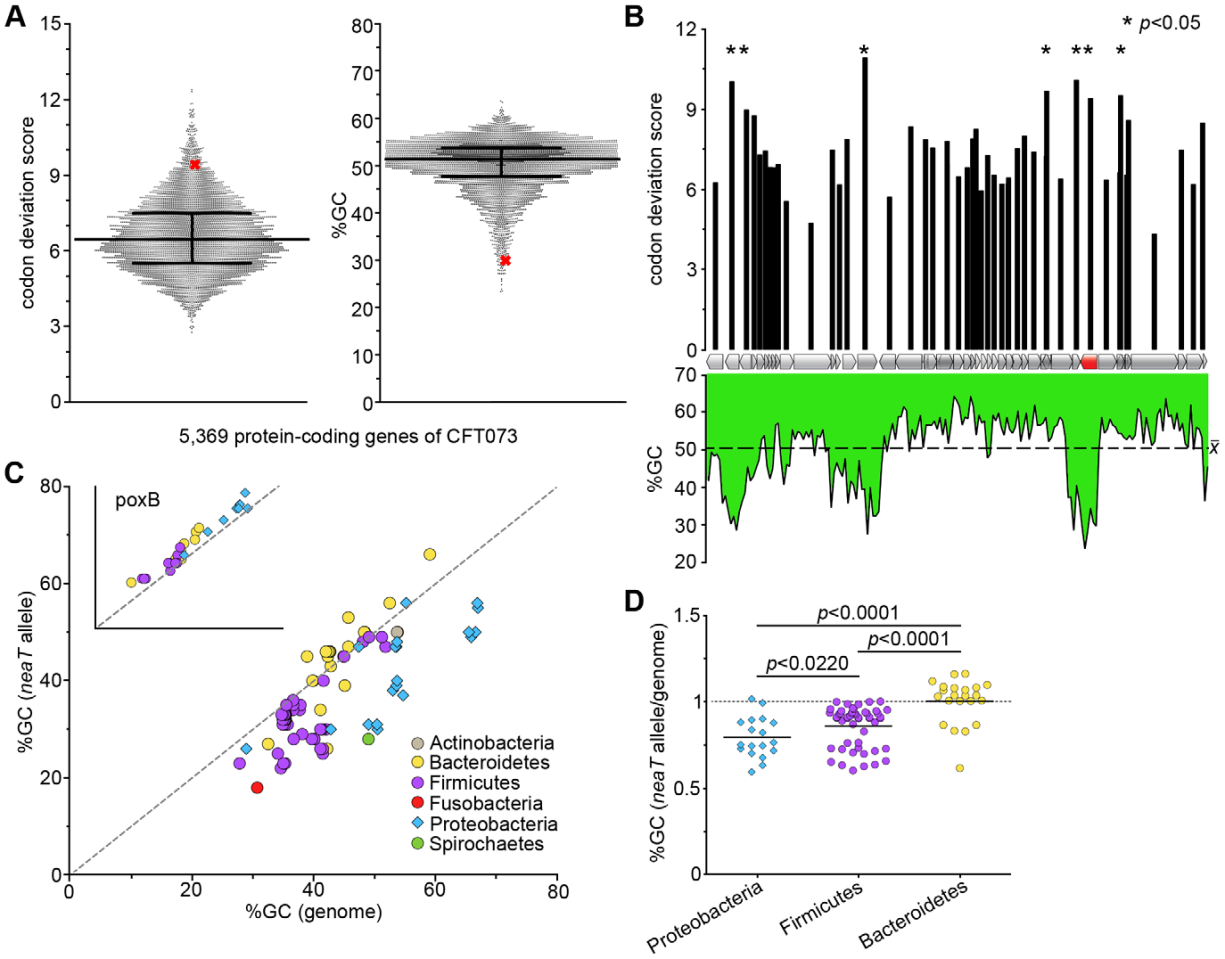
*neaT* is maintained in an un-ameliorated state. (A) Left: distribution of codon deviation scores assigned to the 5,369 protein-coding genes of CFT073. Right: distribution of %GC content of each protein-coding gene of CFT073. Bar and whiskers indicate median and interquartile ranges. Red ‘X’ marks position of *neaT* within each distribution. (B) Upper y-axis: bar graph depicting codon deviation score for each φb0847 gene plotted with respect to position within the prophage (x-axis cartoon, with the *neaT* gene highlighted in red.). Lower y-axis: line graph representing fluctuations in %GC content across φb0847 (window = 182 bp, step = 182 bp). (C) %GC content of *neaT* alleles found across phyla (color-coded) plotted against the %GC content of each respective genome (x-axis). Inset shows the same sort of analysis for the *poxB* allele as a comparison. Points falling on the dashed lines represent alleles that are completely ameliorated with respect to their host genomes. (D) Graph shows the ratio of %GC content of *neaT* alleles and total genomic %GC content for individual isolates within the indicated phyla. Bars indicate medians, and *p* values were determined using two-tailed Mann-Whitney *t* tests (*n* = 18 (Proteobacteria), 51 (Firmicutes), 22 (Bacteroidetes)).

If a gene has origins outside its immediate genome, it would carry sequence-level vestiges of its previous host until it adopts the characteristics of the current host—a process known as ‘amelioration’ [27], [58]. Commonly used parameters that distinguish laterally transferred and unameliorated genes are atypical codon usage and guanine-cytosine (G+C) content [57], [59], [60]. We analyzed these features of *neaT* in the context of the CFT073 genome and φb0847 prophage. Using all 5,369 protein-coding genes of CFT073, the frequency with which specific codons are used for each amino acid was calculated (Table S5). Each gene was then assigned a ‘codon deviation score’ representing how often it uses atypical codons (Methods and Table S6). Scoring correlates with conformity; genes scoring low have a more typical codon usage, whereas poorly conformed genes score high. This analysis shows that *neaT* possesses a significantly abnormal codon usage compared to the rest of the CFT073 genome (*p* = 0.0260) (Figure 6A, left panel). The *neaT* gene was also observed to be G+C-poor (29.84%), making it a significant outlier from the CFT073 genome-wide median of 51.5% (*p* = 0.0001) (Figure 6A, right panel). We also analyzed the codon deviation score (Figure 6B, upper axis) and nucleotide composition (Figure 6B, lower axis) of *neaT* with respect to the genome of φb0847. Most genes withinφb0847 conform to the codon usage and G+C content of CFT073. This is expected for a parasite that has been co-evolving with proteobacterial hosts over an extensive period of evolutionary time [59]. Thus, the aberrant codon usage and nucleotide composition of *neaT* is not simply an inherited trait of φb0847. Because of its relatively low G+C content and poorly conformed codon usage, we conclude that *neaT* is a relatively recent acquisition by both φb0847 and the genome of CFT073.

To determine if the apparently unameliorated state of *neaT* in CFT073 is unique or if it is hinting at a more widespread phenomenon, we plotted the G+C content of a representative subset of *neaT* homologues identified in Figure 5 against the G+C content of their respective genomes (Figure 6C). As a control, we also plotted the G+C content of *poxB*, which encodes the metabolic enzyme pyruvate oxidase and exists in an ameliorated state within several phyla (Figure 6C, inset). Most proteobacterial *neaT* genes were significantly less ameliorated than those found in the genomes of Bacteroidetes and many Firmicutes (Figure 6C and D). Interestingly, even though Firmicutes genomes generally have a low G+C content, *neaT*-like genes within this lineage are still relatively G+C-poor, at least in a major fraction of Firmicute species (Figure 6C). Cumulatively, these results indicate that, at least among the three phyla compared here, *neaT*-like genes have likely been associated with Bacteroidetes the longest, whereas acquisition by the Proteobacteria was a more recent event.

## Discussion

### Summary and impact of findings

Presented here are the results from a screen conducted using the ExPEC isolate CFT073 and a high-throughput zebrafish surrogate host model of infection. We screened GIs for novel virulence genes, which were expected to have a history of lateral gene transfer. Three loci within the P2-like prophage φb0847 were found to contribute to the virulence of CFT073 during systemic infection. A previously uncharacterized gene—designated here as *neaT*—was discovered to augment the virulence capacity of CFT073, independent of other prophage components (Figure 3D). We demonstrated that *neaT* is conditionally required for maximal fitness during bacteremic infections of both zebrafish and mice, suggesting that CFT073 has potentially co-opted this phage-borne gene for specific virulence behaviors. By tracing the evolutionary history of the *neaT* gene, we found that it is relatively rare and has sequence-based features suggesting that it was recently absorbed into the proteobacterial supraspecies pan-genome. Signs of its novelty are typified by high allelic variance—possibly a result of multiple entries into the Proteobacteria lineage via phage—and its mostly unameliorated state within proteobacterial genomes.

We also investigated the putative function(s) of NeaT *in vitro*. The NeaT protein shares homology with several characterized acyltransferases encoded within a variety of non-*E. coli* genomes. These putative membrane-localized enzymes can modify components of the bacterial cell wall, particularly peptidoglycan [61], [62], [63], [64]. Alteration of this macromolecule can often provide bacterial pathogens with protection from host antimicrobial peptides and enzymes such as lysozyme. However, deletion of *neaT* had no effect on the sensitivity of CFT073 to lysozyme, the antimicrobial cationic peptide polymyxin B, or antibacterial factors present in human serum (see accompanying supplemental Text S1). Interestingly, expression of *neaT* did alter the behavior of CFT073 in swarming assays and induced bacterial aggregation on swim plates (Figure S5A–C, Text S1)—phenotypes that may be attributable to NeaT-mediated modification of components within the bacterial envelope. We also found that expression of recombinant NeaT can inhibit production of surface structures like curli and cellulose in some strain backgrounds (Fig. S5D–E, Text S1), supporting the notion that NeaT can affect salient properties of the bacterial surface and thereby alter bacterial group behavior.

The apparent capacity of NeaT to modulate bacterial aggregation (Fig. S5C) is especially intriguing in light of a recent work demonstrating that aggregate formation can promote bacterial survival within the bloodstream of infected mice [65]. Building on these observations, we found that expression of the *neaT* gene from a low copy number plasmid significantly decreased the capacity of CFT073 to associate with murine macrophages, suggesting that NeaT serves as an immune evasion factor (Fig. S6). The specific mechanism(s) by which NeaT promotes bacterial fitness during systemic infections, as well as the environmental cues that control *neaT* expression, require further investigation. As it stands, this work contributes to the idea that ExPEC isolates do not all share the same set of virulence factors, which are likely dictated by the distinct evolutionary trajectory and particular niche tropism of each strain.

### *neaT*-based models for evolution of laterally acquired genes

Our analysis defines *neaT* as a recently acquired locus of the Proteobacteria. Evidence for this is drawn from its discordant conservation, abnormal codon usage, and low G+C content. In large part, the unameliorated state of *neaT*-like genes in Proteobacteria and Firmicutes genomes suggests that there is a general phenomenon accounting for its relative A+T-rich composition beyond having originated in an A+T-rich genome, as previously suggested [59]. We posit that the observed A+T-richness of laterally transferred genes can be, to some extent, accounted for by an ‘exploratory mechanism’ [16]. Upon introgression of a foreign gene, its retention depends on its adaption to the host's genetic and cellular machinery, a process that can take several millions of years [66]. During this time the gene may fall under relaxed selection whereby mutations accrue until a beneficial allele is ‘discovered’ and acted upon by selection. Connecting relaxed selection to reduced G+C content is the observation that there is a universal mutation bias for G/C to A/T transitions in bacterial genomes [67], [68], [69]. It then follows that immediately after a gene is acquired, it will initially accumulate A+T-rich character until a selectable version is ameliorated. From the findings presented here, we speculate that the *neaT* variant in CFT073 is an example of a newfound allele that is being used to promote bacterial fitness in pathogenic contexts.

Arguably, *neaT* may represent an ancient gene that has simply failed to fix within the proteobacterial lineage. Therefore, an alternative hypothesis is that the conditional requirement for *neaT* by CFT073 within different environments may have driven its current evolved state. We observed in two vertebrate model systems that *neaT* contributes significantly to pathogen fitness primarily during systemic infections. Considering the ecology of many bacterial pathogens, a question often left unaddressed is: what are the evolutionary forces that act on niche-specific genes in the absence of selective pressure? Particularly for *E. coli*, which has a complex multi-niche life cycle, the evolutionary consequences resulting from time outside selective environments on genes like *neaT* are not clear.

Work directly addressing this question is scarce. However, insight into this issue is provided by findings that genes under relaxed constraint have increased variance at the sequence level [17], [70], [71], [72], [73]. In contrast to relaxed selection, which occurs when purifying selection is alleviated, as discussed above, ‘relaxed constraint’ refers to a limitation in the exposure of a particular gene to selection. For example, eukaryotic genes with expression patterns that are sex-restricted are effectively ‘hidden’ from selection in half of the population. This is the case for the *Drosophila spp.* maternal-effect gene *bicoid*, which is maternally-restricted and critical for the embryonic development of fruit flies [70]. The *bicoid* gene was found to have a 2-fold higher heterozygosity compared to zygotically-expressed genes. Similarly, genes with caste-biased expression (*i.e.*, queen versus worker) in the social insects *Solenopsis invicta* (fire ant) and *Apis mellifera* (honey bee) were shown to be evolving more rapidly than genes expressed among all castes [71]. For both of these situations, the higher mutation rate observed for contextually expressed genes was concluded to be due to relaxed constraint. Further investigation into the exploratory mechanism and relaxed constraint hypotheses of *neaT* evolution is required and must be considered in parallel with other processes and factors, including, for example, the susceptibility of laterally transferred genes to endogenous restriction enzymes [59].

### ExPEC individuality and virulence

There exists an enormous amount of genetic heterogeneity among Eubacteria lineages. Genome sequencing and bioinformatic analyses have underscored this extensively. Perhaps the most intriguing aspect of this diversity is that even closely related members of the same species can differ greatly with respect to their gene contents. Strikingly, any two *E. coli* genomes can differ by up to 20–30% of their respective gene contents—in sharp contrast to the relatively minor difference of 1% that exists between, for example, the mouse and human species [32], [74]. Decades worth of epidemiological and experimental studies have focused on the identification of genes that define the pathogenic behavior of ExPEC [7], [32]. However, it appears that a single, ubiquitous genetic identifier of ExPEC, such as a gene encoding a particular toxin or adhesin, does not exist and, rather, what actually binds these pathogens is more qualitative and multigenic in nature [32], [75].

In support of this view, we recently demonstrated that the toxin α-hemolysin, shared among many ExPEC isolates, is differentially required for virulence depending on strain background [40]. Similarly, we found that the pathogenicity of particular ExPEC isolates depends on another toxin, cytotoxic necrotizing factor, while other equally virulent strains naturally lack this gene. Coupled with the work presented here, these observations suggest that there exists a spectrum of only partially overlapping virulence gene requirements among ExPEC, reflecting the idea that these pathogens have emerged from distinct evolutionary trajectories driven by LGT [76], [77]. Accordingly, we found that the expression of NeaT from plasmid pGEN-*neaT* ^Pnative^ in other *E. coli* strains, including Nissle 1917 (gut isolate), F11 (cystitis isolate), and S88 (meningitis isolate), did not augment virulence in the zebrafish infection model (data not shown). These findings suggest that the ability of a rare gene like *neaT* to affect fitness and virulence is dependent upon the genetic background of individual bacterial strains. The beneficial effects of *neaT*, and its potential to sweep through bacterial populations, is therefore likely linked to the presence, or coordinate acquisition, of other as-yet undefined bacterial factor(s). The identification, characterization, and continued monitoring of rare genes like *neaT* will be important to our understanding of ExPEC evolution. As a case in point, we note that the *sasX* gene, originally defined as *rare* among strains of methicillin resistant *Staphylococcus aureus* (MRSA), increased in prevalence among MRSA isolates between 2003 and 2011 and is now considered an emerging virulence determinant [78]. Interestingly, like *neaT*, *sasX* is also maintained within a prophage and can affect bacterial interactions with phagocytes. At this point, it is difficult to predict if *neaT* will sweep ExPEC populations in the future, but work presented here along with recent findings concerning *sasX* underscore how laterally acquired genes can alter the virulence potential of bacterial pathogens, continually challenging the development of broad spectrum therapeutics.

Going forward, as we continue to characterize the composition of pan-genomic elements of ExPEC and other pathogens, it will be important to consider the evolutionary context of their virulence genes. Identifying the spatial and temporal parameters that govern the lateral acquisition of virulence genes from distant lineages will need to be reconciled. Genome compatibility (codon and tRNA usage) and ecology are thought to be influential in the success of LGT events between bacteria [79], [80], [81]. In light of this, several interesting questions arise. How did *neaT* come to be in the proteobacterial gene pool? How does residence of *neaT* within a prophage impact its evolution? What conditions fostered the assimilation of *neaT* into the virulence regulon of its host? Using *neaT* as a stepping-stone, it will be informative to resolve the amount of strain-specific innovation that goes into producing and fine-tuning pathogen genomes. By understanding the mechanisms of chromosome assembly and the sources of individual genetic components, unrealized patterns may emerge that could prove useful for future diagnostics and disease mitigation.

## Methods

### Ethics statement

Animals used in this study were handled in accordance with IACUC protocols approved at either the University of Utah or the University of Michigan Medical School following standard guidelines as described at www.zfin.org and in the Guide for the Care and Use of Laboratory Animals, 8th Edition [55], [82].

### Bacterial strains and plasmids

All bacterial strains and plasmids used in this study are listed in Table 3. Unless specified otherwise, bacteria were cultured statically at 37°C for 24 h in 20 ml of a defined M9 minimal medium (6 g/l Na_2_HPO_4_, 3 g/l KH_2_PO_4_, 1 g/l NH_4_Cl, 0.5 g/l NaCl, 1 mM MgSO_4_, 0.1 mM CaCl_2_, 0.1% glucose, 0.0025% nicotinic acid, 0.2% casein amino acids, and 16.5 mg/ml thiamine in H_2_O). Antibiotics (kanamycin or ampicillin) were added to the growth medium when necessary to maintain recombinant plasmids or select for mutants.

**Table 3:** Bacterial strains and plasmids.

Strain or Plasmid	Description	Reference
*E. coli*		
CFT073	ExPEC (urosepsis isolate, O6:K2:H1)	[26]
Nissle 1917	Probiotic (gut isolate, O6:K5:H1)	[86]
HS	Commensal (gut isolate, O9)	[87]
Plasmids		
pKM208	Encodes IPTG-inducible lambda Red recombinase; Amp^r^	[88]
pKD4	Template source for kanamycin resistance cassette;Kan^r^	[84]
pGEN-mcs	High retention vector with an empty multiple cloning site; Amp^r^	[85]
pGEN-*neaT* ^Pnative^	pGEN-mcs containing a natively controled *neaT* variant; Amp^r^	This study
pGEN-*neaT* ^P*lac*^	pGEN-mcs containing a *neaT* variant constitutively expressed from a leaky *lac* promoter; Amp^r^	This study
Recombinant strains		
CFT073/pKM208	CFT073 with pKM208; Amp^r^	This study
CFT073Δφb0847	CFT073 φb0847::*kan*	[45], this study
CFT073Δ1–2	CFT073 1–2::*kan* (locus tags c0932 through c0945)	This study
CFT073Δ2–3	CFT073 2–3::*kan* (locus tags c0946 through c0962)	This study
CFT073Δ3–4	CFT073 3–4::*kan* (locus tags c0963 through c0978)	This study
CFT073Δ*neaT*	CFT073 *neaT*::*kan* (locus tag c0970)	This study
CFT073Δ*yfdK*	CFT073 *yfdK*::*kan* (locus tag c0969)	This study
CFT073Δ*FI-D*	CFT073 *FI-D*::*kan* (locus tags c0971 through c0978)	This study
CFT073/pGEN-mcs	CFT073 with pGEN-mcs (empty vector); Amp^r^	This study
CFT073/pGEN-*neaT* ^P*lac*^	CFT073 with pGEN-*neaT* ^P*lac*^; Amp^r^	This study
CFT073Δ*neaT*/pGEN-mcs	CFT073Δ*neaT* with pGEN-mcs (empty vector); Amp^r^, Kan^r^	This study
CFT073Δ*neaT*/pGEN-*neaT* ^Pnative^	CFT073Δ*neaT* with pGEN-*neaT* ^Pnative^; Amp^r^, Kan^r^	This study
CFT073Δ*neaT*/pGEN-*neaT* ^P*lac*^	CFT073Δ*neaT* with pGEN-*neaT* ^P*lac*^; Amp^r^, Kan^r^	This study
CFT073Δφb0847/pGEN-mcs	CFT073Δφb0847 with pGEN-mcs (empty vector); Amp^r^, Kan^r^	This study
CFT073Δφb0847/pGEN-*neaT* ^Pnative^	CFT073Δφb0847 with pGEN-*neaT* ^Pnative^; Amp^r^, Kan^r^	This study
Nissle 1917/pGEN-mcs	Nissle 1917 with pGEN-mcs (empty vector); Amp^r^	This study
Nissle 1917/pGEN-*neaT* ^Pnative^	Nissle 1917 with pGEN-*neaT* ^Pnative^; Amp^r^	This study
Nissle 1917/pGEN-*neaT* ^P*lac*^	Nissle 1917 with pGEN-*neaT* ^P*lac*^; Amp^r^	This study
HS/pGEN-mcs	HS with pGEN-mcs (empty vector); Amp^r^	This study
HS/pGEN-*neaT* ^Pnative^	HS with pGEN-*neaT* ^Pnative^; Amp^r^	This study
HS/pGEN-*neaT* ^P*lac*^	HS with pGEN-*neaT* ^P*lac*^; Amp^r^	This study

Targeted gene knockouts were generated in the ExPEC isolate CFT073 using the lambda Red-mediated linear transformation system [83], [84]. Briefly, a kanamycin resistance cassette was amplified using polymerase chain reaction (PCR) from pKD4 with 40-base pair overhangs specific to the 5′ and 3′ ends of each targeted locus. PCR products were introduced via electroporation into CFT073 carrying pKM208, which encodes an IPTG (isopropyl-β-D-thiogalactopyranoside)-inducible lambda red recombinase. Knockouts were confirmed by PCR. Primer sets used are listed in Table S8.

Cloning and construction of *neaT* expression constructs were done using standard molecular techniques employing the high-retention plasmid pGEN-mcs [85]. For native regulation, *neaT* (locus tag: c0970), plus 211 bp of upstream sequences, were amplified from the chromosome of CFT073 and TA-cloned into pCR2.1-TOPO vector per manufacture's protocol (Invitrogen). Subsequently, the cloned fragment was isolated using BamHI and NotI restriction enzymes (New England Biosciences) and ligated into pGEN-mcs using the same sites, yielding pGEN-*neaT* ^Pnative^. For construction of pGEN-*neaT* ^P*lac*^, a synthetic ribosome binding sequence was introduced upstream of *neaT* within the 5′ PCR primer, and the resulting PCR product was ligated via an engineered NdeI restriction site with the *lac* promoter amplified from pGFPmut3.1 (Clonetech). The ligated P*lac*-*neaT* product was amplified and TA-cloned into the pCR2.1-TOPO vector. Using BamHI and NcoI restrictions sites, the P*lac* controlled *neaT* variant was then sub-cloned into pGEN-mcs. All experiments involving pGEN-*neaT* ^P*lac*^ were performed without IPTG induction. Primer sequences used to generate these plasmids are listed in Table S8.

### Zebrafish embryos

*AB wild-type zebrafish embryos were collected from a laboratory-breeding colony that was maintained on a 14-h/10-h light/dark cycle. Embryos were grown at 28.5°C in E3 medium (5 mM NaCl, 0.17 mM KCl, 0.4 mM CaCl_2_, 0.16 mM MgSO_4_) containing 0.000016% methylene blue as an anti-fungal agent.

### Infection of zebrafish embryos

One ml from each 24 h bacterial culture was pelleted, washed once with 1 ml sterile PBS (Hyclone) and re-suspended in 1 ml PBS to obtain appropriate bacterial densities for microinjection. Prior to injection, 48 hpf embryos were manually dechorionated, briefly anesthetized using 0.77 mM ethyl 3- aminobenzoate methanesulfonate salt (tricaine) (Sigma-Aldrich), and embedded in 0.8% low-melt agarose (MO BIO Laboratories) without tricaine. Approximately 1 nl of bacteria was injected directly into the pericardial cavity or the blood via the circulation valley located ventral to the yolk sac using a YOU-1 micromanipulator (Narishige), a Narishige IM-200 microinjector, and a JUN-AIR model 3-compressor setup. For each experiment, average CFU introduced per injection were determined by adding 10 drops of each inoculum into 1 ml 0.7% NaCl, which was then serially diluted and plated on Luria-Bertani (LB) agar plates. For co-challenge experiments, input doses were plated on LB agar+/−kanamycin (50 µg/ml) to determine relative numbers of the wild type and mutant strains present. After injection, embryos were carefully extracted from the agar and placed individually into wells of a 96-well microtiter plate (Nunc) containing E3 medium lacking both tricaine and methylene blue. For lethality assays, fish were examined at indicated times over the course of a 48 or 72 h period and scored for “death”, defined here as the complete absence of heart rhythm and blood flow. Survival graphs depict total pooled results from two or more independent experiments in which groups of 10 to 20 embryos were injected. To quantify bacterial numbers during the course of co-challenge experiments, embryos were homogenized at the indicated time points in 500 µL PBS containing 0.5% Triton X-100 using a mechanical PRO 250 homogenizer (PRO Scientific). Homogenates were then serially diluted and plated on LB agar+/−kanamycin (50 µg/ml) to determine relative numbers of wild type and mutant bacteria.

### Mouse infections

For co-challenge during urinary tract infection, seven- to nine-week old female CBA/J mice (Jackson Labs) mice were anesthetized using isoflurane inhalation and inoculated via transurethral catheterization with 50 µl of a 1∶1 wild type to mutant bacterial suspension containing a total of 10^7^ bacteria suspended in PBS. Bladders and kidneys were recovered 3 days later and each was weighed and homogenized in 1 ml containing 0.025% Triton X-100. Homogenates were serially diluted and plated on LB agar+/−kanamycin (50 µg/ml) to determine number of both wild type and mutant bacteria. Mouse experiments were repeated at least twice.

For systemic infections, female CBA/J mice (Jackson Labs) aged 6 to 8 weeks were restrained using a Universal Restrainer (Braintree Scientific, Braintree, MA) and inoculated via the tail vein over a 30 s period with a 100 µl bacterial suspension, delivering 10^6^ CFU/mouse. The inoculum was prepared by re-suspending overnight cultures in PBS and diluting them to 1×10^7^ CFU/ml. For co-challenges, wild type and mutant suspensions were mixed 1∶1 before inoculation. Perfusion was performed on euthanized animals by cutting a small hole in the right cardiac ventricle and infusing the left ventricle slowly with 40 ml 0.9% sterile saline before organ removal. Blanching of the organs occurred with the first 20 ml of sterile saline. Excised spleens and livers were homogenized in 3 ml PBS using a mechanical homogenizer (Omni International, Marietta, GA), and homogenates were plated using an Autoplate 4000 (Spiral Biotech, Norwood, MA) onto LB agar+/−kanamycin (50 µg/ml) to differentiate wild type and mutant strains.

### Statistical analysis of zebrafish and mouse infections

Kaplan-Meier survival and scatter plots were generated using GraphPad Prism 5. For Kaplan-Meier survival plots (independent challenges), the log-rank (Mantel-Cox) test was used to determine statistical differences between datasets. For competitive assays (co-challenges), numbers of wild type and mutant bacteria present in the inoculum and recovered from host tissues were determined as described above and a competitive index was calculated using the following equation where wt represents numbers wild type bacteria:
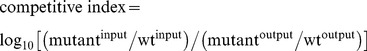
Negative values obtained using the competitive index equation indicate a reduction in mutant fitness. To determine statistical significance, the Wilcoxon signed-rank test (with a hypothetical value of 0) on log-transformed competitive index values was used for co-challenges and the two-tailed Mann-Whitney statistical analysis was performed to determine significant differences between samples in non-competitive assays.

### Bioinformatic analyses

#### Homology searches and phyletic enrichment of homologue sets

A custom database of 165 genomes and associated plasmids was assembled using the compilation of protein coding genes (.faa files downloaded from ftp://ftp.ncbi.nih.gov/genomes/Bacteria/) of each isolate listed in Table S3. BLASTp (v. 2.2.20, [56]) was used to run bidirectional protein alignments between the φb0847 genome and the database to identify homologues. Two sequences were considered homologous if they aligned along >50% of their lengths with an E value of <10^−6^. To identify phyletic enrichment, sets of non-paralogous homologues for each φb0847 gene were analyzed for relative contributions made by each phylum. Then, based on the number genes in each homologue set, the same number of genomes was randomly sampled from the genome list in Table S3. In this way, we could determine the significance of the phyletic contributions to each homologue set that was observed compared to a theoretical random sampling. With custom software written in Python using SciPy, p values were generated from a Z score. Standard scores were calculated using the equation below, where x is the observed proportion contributed by a single phylum, μ is the theoretical average contribution by the same phylum (n = 1000 random samplings), and σ is the standard deviation: z = (x−μ)/σ

Sequences used for comparisons between the GC content of *neaT* from CFT073 and homologues in other bacteria (see Figure 6C) were retrieved manually from NCBI for downstream analysis. Genome GC compositions were obtained from NCBI Genomes (http://www.ncbi.nlm.nih.gov/genomes/MICROBES/microbial_taxtree.html)

#### Nucleotide composition analysis

For nucleotide composition analysis, the nucleotide sequences of protein coding genes of CFT073 (.ffn files downloaded from ftp://ftp.ncbi.nih.gov/genomes/Bacteria/Escherichia_coli_CFT073_uid57915/) were used to calculate codon deviation scores and GC content using custom software written in Python with SciPy or NumPy. For codon deviation scores, genome-wide protein coding nucleotide sequences were analyzed for codon usage frequencies on a per amino acid basis. The resulting table (Table S5) was then used to determine differences between specific codon frequencies contained within a particular gene and the genome-wide frequency. The absolute values of differences in frequency were summed over a single gene to obtain the final codon deviation score. Statistical significance was determined by Z-score analysis using the genome-wide mean codon deviation score and standard deviation. Table S6 lists all deviation scores and *p* values for the CFT073 genome. GC content of genes was determined by counting the proportion of guanines and cytosines over the length of a given locus, and Z-score analysis was again implemented to determine the position of each gene within the genome-wide distribution (Table S7).

## Supporting Information

Figure S1 **Expression of the neaT gene in various mutant backgrounds.** RNA was extracted from the indicated strains after overnight growth in M9 medium and used to generate cDNA libraries by reverse transcription (+RT). To control for genomic DNA contamination, a set of samples was prepared in parallel without reverse transcriptase (−RT). Wild type CFT073, CFT073ΔneaT, CFT073ΔyfdK, and CFT073ΔFI-D were used to determine the relative expression levels of neaT in each genetic background. Three µg of each cDNA library was used as a template for PCR amplification (30 cycles) of an internal 218 bp fragment of neaT. Equal amounts of each PCR reaction were resolved using 1% agarose gels.
(TIF)

Figure S2 **Determination of** ***in vivo*** **lateral transfer of the** ***neaT*** **gene.** Zebrafish were inoculated with a one-to-one mixture of wt CFT073 and CFT073**Δ**neaT. Infections progressed for ∼12 h post-inoculation prior to homogenization and recovery of bacteria by plating on LB agar+/−kanamycin. Bacterial colonies recovered from 5 separate fish were used for colony PCR to detect presence of either the kanamycin resistance gene (lane 1 control, ∼1,500 bp) or neaT (lane 2 control, 218 bp internal fragment). No double positive colonies were detected. Primers used to amplify the kanamycin gene are specific to the priming regions of the pKD4 template plasmid. neaT was amplified using neaT RT forward/reverse (Table S8).
(TIF)

Figure S3 **Plasmid-based** ***neaT*** **expression analysis.** RNA was extracted from the indicated strains after overnight growth in LB broth and used to generate cDNA libraries by reverse transcription (+RT). To control for genomic DNA contamination, a set of samples was prepared in parallel without reverse transcriptase (−RT). Wild type (WT) CFT073 or HS were used to reference basal neaT message levels. CFT073ΔneaT or HS carrying pGEN-mcs (empty vector, EV), pGEN-neaT^Pnative^ (native promoter, NP), or pGEN-neaT^Plac^ (over-expressing, OE) were used to determine the relative expression levels of pGEN-neaT variants in each genetic background. Three µg of each cDNA library was used as a template for PCR amplification (28 cycles) of an internal 218 bp fragment of neaT. Equal amounts of each PCR reaction were resolved using 1% agarose gels. Graph shows average levels of neaT transcripts ± SD normalized to 16S rRNA (not shown). Data are presented relative to WT CFT073, n = 3.
(TIF)

Figure S4 **Survey of clinical isolates for presence of the** ***neaT*** **gene.** Various clinical E. coli isolates were surveyed for presence of the neaT gene using polymerase chain reaction. Primers used in (A) amplified a 218 bp region internal to neaT (Table S8). (B) Shows amplification of the 16s ribosomal RNA gene as a control. Isolates are described as: strain (clinical disease presentation).
(TIF)

Figure S5 ***neaT*** **contributes to multicellular behaviors.** (A) Swarm motility of wild type (wt) CFT073 and its mutant derivatives on 0.25% Eiken agar plates following overnight incubation at 37°C. (B) Complementation of swarm defect of CFT073Δ*neaT* by introduction of pGEN-*neaT* ^P*lac*^. The empty vector pGEN-mcs and pGEN-*neaT* ^Pnative^ did not complement the Δ*neaT* mutant. (C) Swim motility of indicated CFT073 derivatives following incubations at 37°C for times indicated. Red arrows indicate advancing swim fronts and insets show magnified bright field images of the center region of each plate. (D) Images of single Nissle 1917 colonies carrying pGEN-mcs or pGEN-*neaT* ^P*lac*^ grown for 48 h at 37°C on agar plates containing 0.001% Congo red dye to stain curli fibers. (E) Streaks of Nissle 1917 carrying pGEN-mcs, pGEN-*neaT* ^Pnative^, or pGEN-*neaT* ^P*lac*^ grown overnight at 37°C on 1.2% LB agar containing 50 µg/ml Fluorescent Brightener 28 to visulalize cellulose production. Image was captured under ultraviolet light.
(TIF)

Figure S6 **NeaT limits bacterial interactions with murine macrophages.** (Left) The indicated bacterial strains were added to bone marrow derived macrophage (BMDM) monolayers at a multiplicity of infection of 10. After a 1-h incubation at 37C, total viable bacteria remaining in the wells were enumerated. (Right) Alternatively, monolayers were washed at the 1-h time point with PBS, prior to lysis, in order to determine numbers of macrophage-associated bacteria. Bars represent the means ± SD of three independent experiments performed in triplicate. *p<0.05, **p<0.01; as determined by Student's t test.
(TIF)

Table S1 **Summary of results obtained from initial GI screen using different dose ranges.**
(XLSX)

Table S2 **Summary of inconclusive** ***in vitro*** **experiments.**
(XLSX)

Table S3 **List of strains contained within the custom 165 genome database.**
(XLSX)

Table S4 **List of strains/genomes from the 165 genome database that contain at least one** ***neaT*** **homologue.**
(XLSX)

Table S5 **Codon usage frequency for each amino acid based on the 5,369 protein-coding genes of CFT073.**
(XLSX)

Table S6 **Codon deviation score for each gene in CFT073.**
(XLSX)

Table S7 **GC content for each gene in CFT073.**
(XLSX)

Table S8 **Primers used in this study to generate recombinant strains and plasmids.**
(XLSX)

Text S1 **Expression of NeaT may alter bacterial group behavior.**
(DOCX)

Text S2 **Supporting methods used in Text S1, Figure S5 and S6.**
(DOCX)
