# Supplementary figures and images for: A Phyletically Rare Gene Promotes the Niche-specific Fitness of an E. coli Pathogen during Bacteremia
Source: PLoS Pathog. 2013 Feb 14;9(2):e1003175. doi: 10.1371/journal.ppat.1003175 (PMC3573123; doi:10.1371/journal.ppat.1003175)

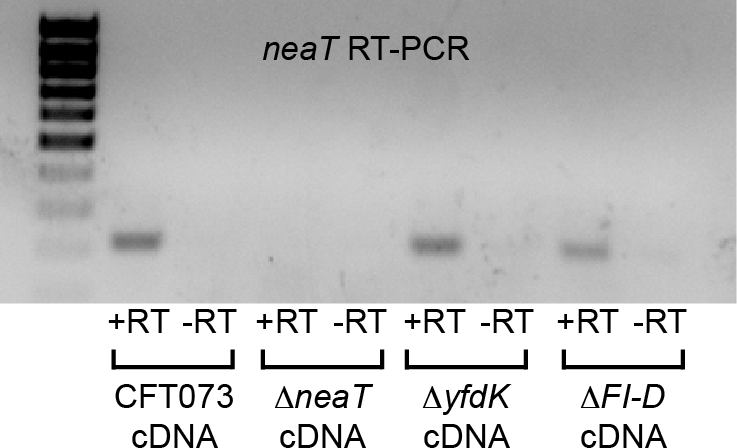

Supplement: Figure S1 — Expression of the neaT gene in various mutant backgrounds. RNA was extracted from the indicated strains after overnight growth in M9 medium and used to generate cDNA libraries by reverse transcription (+RT). To control for genomic DNA contamination, a set of samples was prepared in parallel without reverse transcriptase (−RT). Wild type CFT073, CFT073ΔneaT, CFT073ΔyfdK, and CFT073ΔFI-D were used to determine the relative expression levels of neaT in each genetic background. Three µg of each cDNA library was used as a template for PCR amplification (30 cycles) of an internal 218 bp fragment of neaT. Equal amounts of each PCR reaction were resolved using 1% agarose gels. (TIF) [file ppat.1003175.s001.tif]

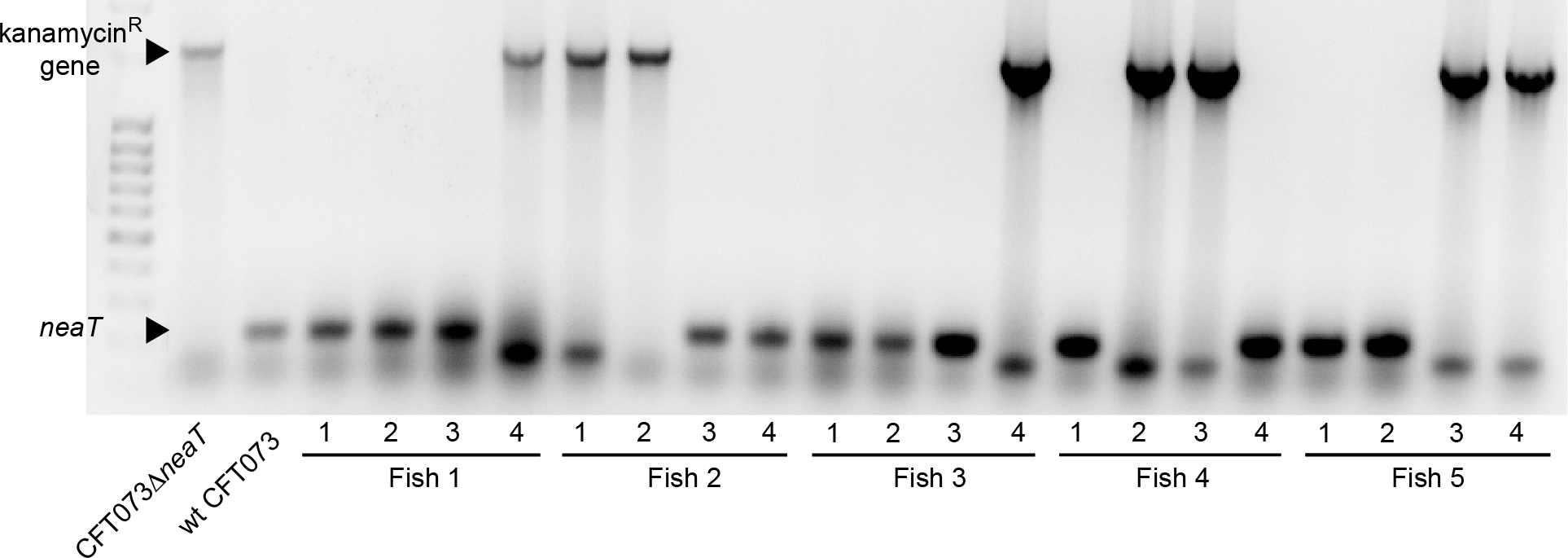

Supplement: Figure S2 — Determination of in vivo lateral transfer of the neaT gene. Zebrafish were inoculated with a one-to-one mixture of wt CFT073 and CFT073ΔneaT. Infections progressed for ∼12 h post-inoculation prior to homogenization and recovery of bacteria by plating on LB agar+/−kanamycin. Bacterial colonies recovered from 5 separate fish were used for colony PCR to detect presence of either the kanamycin resistance gene (lane 1 control, ∼1,500 bp) or neaT (lane 2 control, 218 bp internal fragment). No double positive colonies were detected. Primers used to amplify the kanamycin gene are specific to the priming regions of the pKD4 template plasmid. neaT was amplified using neaT RT forward/reverse (Table S8). (TIF) [file ppat.1003175.s002.tif]

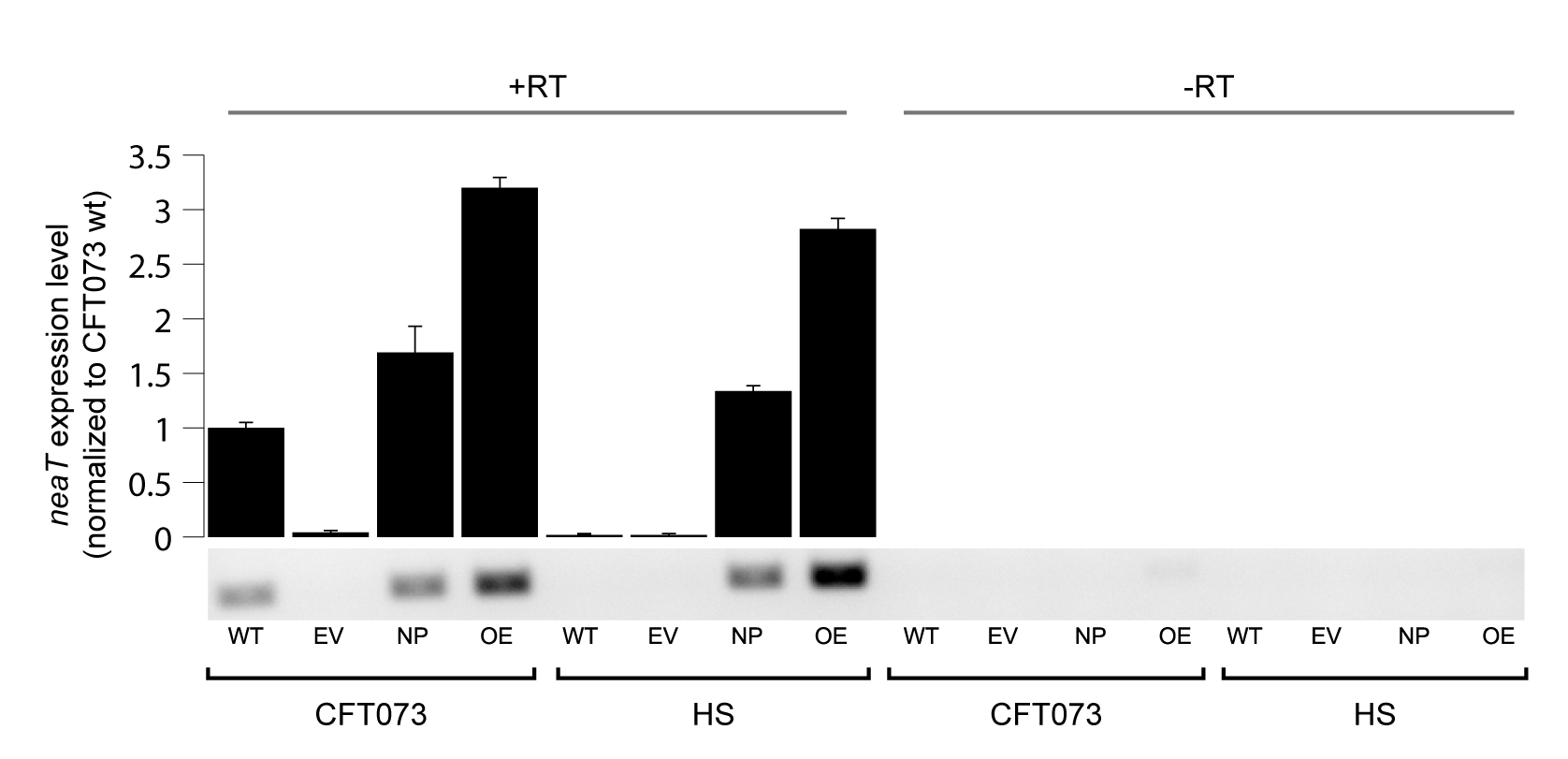

Supplement: Figure S3 — Plasmid-based neaT expression analysis. RNA was extracted from the indicated strains after overnight growth in LB broth and used to generate cDNA libraries by reverse transcription (+RT). To control for genomic DNA contamination, a set of samples was prepared in parallel without reverse transcriptase (−RT). Wild type (WT) CFT073 or HS were used to reference basal neaT message levels. CFT073ΔneaT or HS carrying pGEN-mcs (empty vector, EV), pGEN-neaTPnative (native promoter, NP), or pGEN-neaTPlac (over-expressing, OE) were used to determine the relative expression levels of pGEN-neaT variants in each genetic background. Three µg of each cDNA library was used as a template for PCR amplification (28 cycles) of an internal 218 bp fragment of neaT. Equal amounts of each PCR reaction were resolved using 1% agarose gels. Graph shows average levels of neaT transcripts ± SD normalized to 16S rRNA (not shown). Data are presented relative to WT CFT073, n = 3. (TIF) [file ppat.1003175.s003.tif]

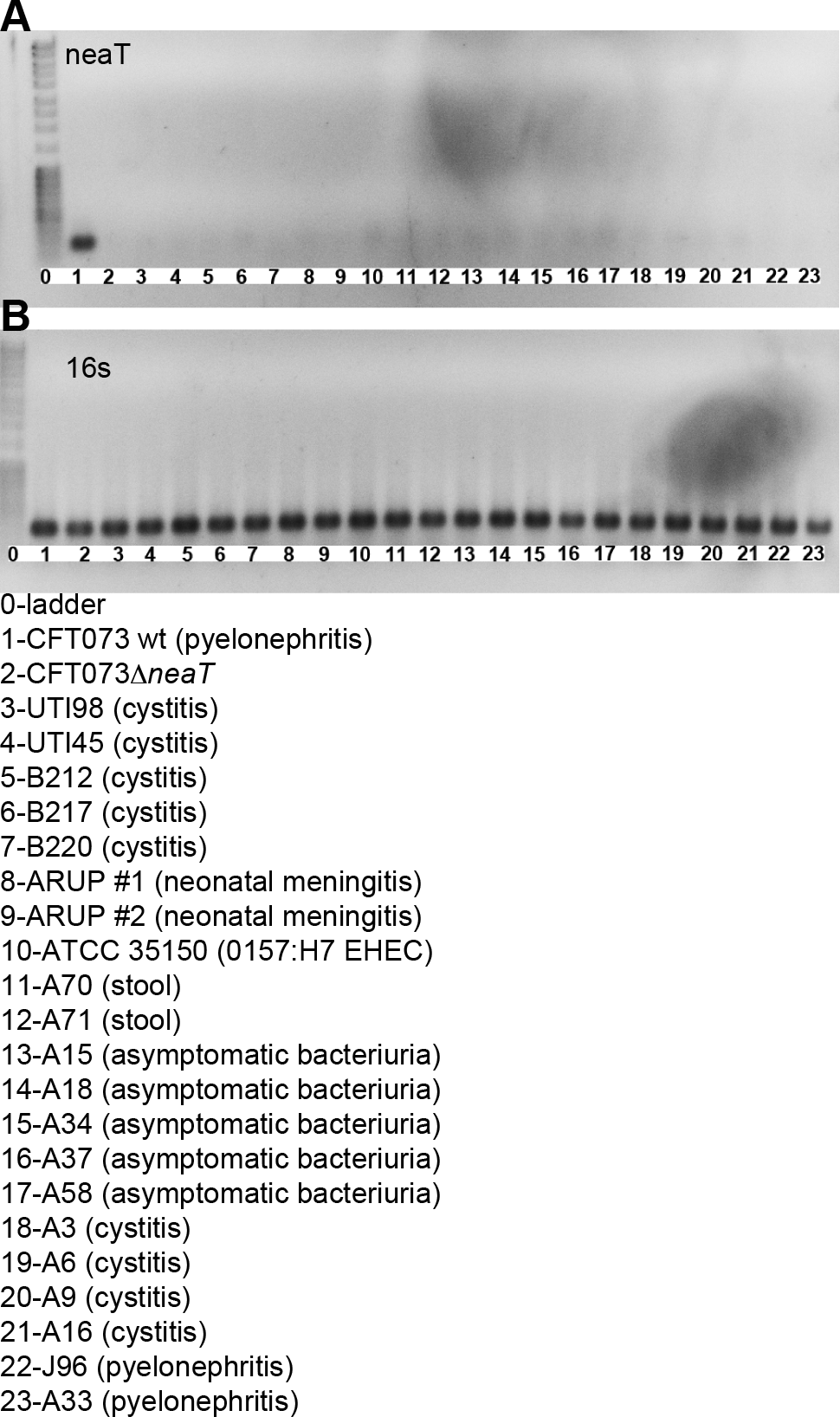

Supplement: Figure S4 — Survey of clinical isolates for presence of the neaT gene. Various clinical E. coli isolates were surveyed for presence of the neaT gene using polymerase chain reaction. Primers used in (A) amplified a 218 bp region internal to neaT (Table S8). (B) Shows amplification of the 16s ribosomal RNA gene as a control. Isolates are described as: strain (clinical disease presentation). (TIF) [file ppat.1003175.s004.tif]

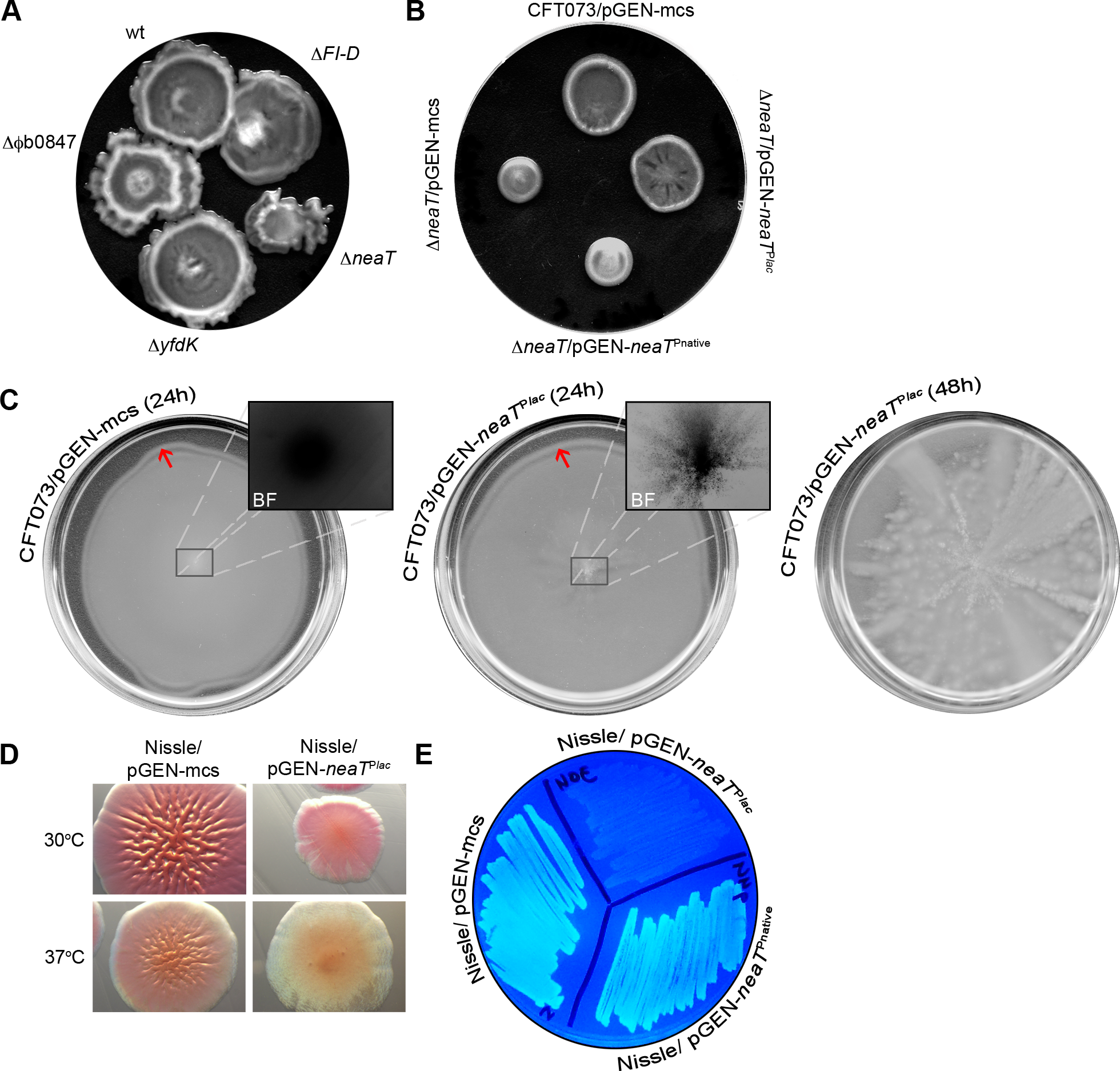

Supplement: Figure S5 — neaT contributes to multicellular behaviors. (A) Swarm motility of wild type (wt) CFT073 and its mutant derivatives on 0.25% Eiken agar plates following overnight incubation at 37°C. (B) Complementation of swarm defect of CFT073ΔneaT by introduction of pGEN-neaT Plac. The empty vector pGEN-mcs and pGEN-neaT Pnative did not complement the ΔneaT mutant. (C) Swim motility of indicated CFT073 derivatives following incubations at 37°C for times indicated. Red arrows indicate advancing swim fronts and insets show magnified bright field images of the center region of each plate. (D) Images of single Nissle 1917 colonies carrying pGEN-mcs or pGEN-neaT Plac grown for 48 h at 37°C on agar plates containing 0.001% Congo red dye to stain curli fibers. (E) Streaks of Nissle 1917 carrying pGEN-mcs, pGEN-neaT Pnative, or pGEN-neaT Plac grown overnight at 37°C on 1.2% LB agar containing 50 µg/ml Fluorescent Brightener 28 to visulalize cellulose production. Image was captured under ultraviolet light. (TIF) [file ppat.1003175.s005.tif]

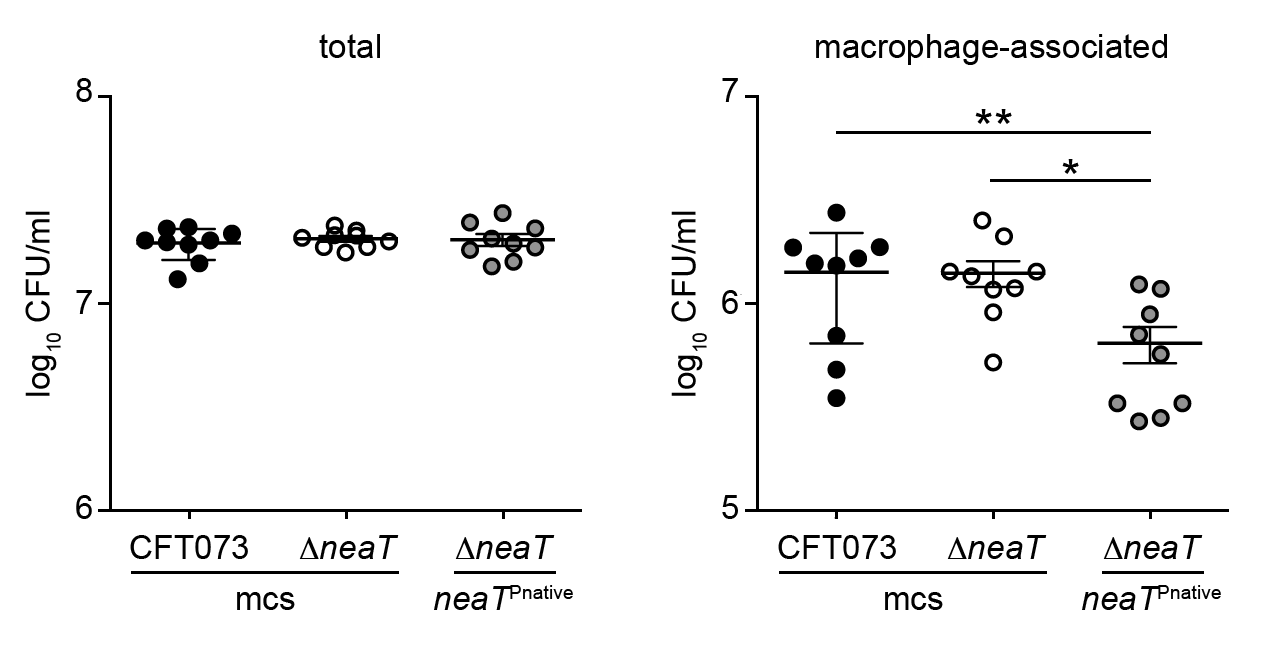

Supplement: Figure S6 — NeaT limits bacterial interactions with murine macrophages. (Left) The indicated bacterial strains were added to bone marrow derived macrophage (BMDM) monolayers at a multiplicity of infection of 10. After a 1-h incubation at 37C, total viable bacteria remaining in the wells were enumerated. (Right) Alternatively, monolayers were washed at the 1-h time point with PBS, prior to lysis, in order to determine numbers of macrophage-associated bacteria. Bars represent the means ± SD of three independent experiments performed in triplicate. *p<0.05, **p<0.01; as determined by Student's t test. (TIF) [file ppat.1003175.s006.tif]
