# Supplementary material for: A Phyletically Rare Gene Promotes the Niche-specific Fitness of an E. coli Pathogen during Bacteremia
Source: PLoS Pathog. 2013 Feb 14;9(2):e1003175. doi: 10.1371/journal.ppat.1003175 (PMC3573123; doi:10.1371/journal.ppat.1003175)
Supplement: Text S1 — Expression of NeaT may alter bacterial group behavior. (DOCX) [file ppat.1003175.s015.docx]

Supplemental Text S1

Expression of NeaT may alter bacterial group behavior

The NeaT protein is a predicted acyltransferase belonging to the Acyltransferase 3 family of proteins (Pfam: PF01757) [1]. This family contains an array of mostly uncharacterized bacterial enzymes that appear to have different but related functions, including modification of peptidoglycan and exopolysaccharide [2,3,4,5]. We were unable to implicate *neaT* in the modification of any cell wall components (Table S2), but we did find that *neaT* can modulate the swarming behavior of CFT073 (Figure S5A). Swarming is a cooperative form of cell motility that occurs when bacteria move across a surface in multicellular waves [6]. This type of motility is dependent on flagella, cell-to-cell contacts, and a surfactant. In *E. coli*, swarming is also regulated by exopolysaccharides [6,7]. CFT073Δ*neaT* was notably impaired in its ability to swarm on agar plates relative to the wild type strain and the Δ*yfdK* and Δ*FI-D* mutants (Figure S5A). Curiously, the Δϕb0847 mutant did not mirror the swarming deficiency of the *neaT* knockout. This indicates that perturbations in swarming were likely not the sole basis for attenuation of the original Δϕb0847 mutant in zebrafish and mice. Furthermore, these data suggest that interplay between *neaT* and other gene products encoded within ϕb0847 can affect normal bacterial processes like swarming. The swarming defect associated with the Δ*neaT* mutant was complemented by the plasmid pGEN-$neaT^{Plac}$, which encodes a constitutively expressed variant of *neaT* driven by a leaky *lac* promoter (Figure S5B and S1). In contrast, pGEN-$neaT^{Pnative}$, which restored virulence to the full ϕb0847 island mutant within the zebrafish host (Figure 3D), failed to rescue the swarming defect of CFT073Δ*neaT* (Figure S5B), suggesting that there are additional

regulatory elements absent from this plasmid that are responsible for plate-associated swarming.

In contrast to swarming, swimming motility is a single cell behavior involving the flagella-dependent movement of bacteria through a liquid medium in response to chemoattractive and repulsive gradients. In plate assays, CFT073Δ*neaT* exhibited no defect in swimming, indicating that its inability to swarm is likely not due to altered flagella function (data not shown). However, in these assays, CFT073 carrying pGEN-*neaT*^{Plac} displayed an unexpected trait. This recombinant strain produced an advancing swim front comparable to that of wild type CFT073 (Figure S5C, red arrows), but the wave of swimming bacteria was less dense and contained large bacterial aggregates that increased in size and number with time (Figure S5C, center and right). This phenotype was readily observed by bright field microscopy where the wild type strain displays an even distribution of bacteria that blocks transmitted light, while CFT073/ pGEN-*neaT*^{Plac} formed punctuated aggregates amidst a thinner layer of cells (Figure S5C, left and center insets). Use of dye exclusion LIVE/DEAD assays revealed that the bacteria within these communities were viable (data not shown). Furthermore, constitutive expression of *neaT* was sufficient to induce this swim-associated aggregation phenotype in other *E. coli* strains, including Nissle 1917, ED1a, S88, and F11, which are all naturally deficient for *neaT* (data not shown). Importantly, the formation of these aggregates required the semi-solid stratum of the agar swim plates. There were no signs of intercellular aggregates when cells carrying pGEN-*neaT*^{Plac} were cultured planktonically during shaking or static conditions. To our knowledge, this is the first time this type of behavior has been observed. However, these patterns of

aggregation resemble, to some extent, the periodicity of surface binding and cluster formation observed with some bacterial strains in response to chemoattractant gradients stimulated by cellular stress [8,9].

Cumulatively, these *in vitro* studies indicate that *neaT* may actuate multicellular behavior, possibly through modification of surface structures like exopolysaccharide or via effects on regulatory cascades. Further support for this conclusion comes from experiments employing agar plates containing Congo red, a dye that stains amyloid-like curli fibers in addition to cellulose [10]. These extracellular polymers can promote the formation of bacterial communities like biofilms. The *neaT* gene does not affect biofilm development by CFT073 (Table S2), and this pathogen does not produce appreciable levels of either curli or cellulose on agar plates. However, the reference gut isolate Nissle 1917 does express these extracellular fibers, exhibiting what is described as the ‘pdar’ (pink, dry, and rough) morphotype at both 30°C and 37°C (Figure S5D) [11]. Upon forced expression of NeaT in Nissle 1917, the pdar morphotype is greatly diminished (Figure S5D). Using Fluorescent Brightener 28 (a Calcofluor-white substitute that binds cellulose and is detectable under ultraviolet light), it was apparent that loss of the pdar morphotype was accompanied by greatly diminished cellulose production (Figure S5E). Thus, *neaT* can affect the expression of factors like cellulose that alter bacterial group behavior, but the specific mechanism(s) by which *neaT* affects the fitness and virulence of CFT073 remain undefined.

References

1. Punta M, Coggill PC, Eberhardt RY, Mistry J, Tate J, et al. (2012) The Pfam protein families database. Nuc Acids Res 40: D290-301.
2. Berck S, Perret X, Quesada-Vincens D, Prome J, Broughton WJ, et al. (1999) NolL of Rhizobium sp. strain NGR234 is required for O-acetyltransferase activity. J Bacteriol 181: 957-964.
3. Bera A, Herbert S, Jakob A, Vollmer W, Gotz F (2005) Why are pathogenic staphylococci so lysozyme resistant? The peptidoglycan O-acetyltransferase OatA is the major determinant for lysozyme resistance of *Staphylococcus aureus*. Mol Microbiol 55: 778-787.
4. Yoshida Y, Yang J, Peaker PE, Kato H, Bush CA, et al. (2008) Molecular and antigenic characterization of a Streptococcus oralis coaggregation receptor polysaccharide by carbohydrate engineering in Streptococcus gordonii. J Biol Chem 283: 12654-12664.
5. Vollmer W (2008) Structural variation in the glycan strands of bacterial peptidoglycan. FEMS Microbiol Rev 32: 287-306.
6. Kearns DB (2010) A field guide to bacterial swarming motility. Nat Rev Microbiol 8: 634-644.
7. Girgis HS, Liu Y, Ryu WS, Tavazoie S (2007) A comprehensive genetic characterization of bacterial motility. PLOS Gen 3: 1644-1660.
8. Mittal N, Budrene EO, Brenner MP, Van Oudenaarden A (2003) Motility of *Escherichia coli* cells in clusters formed by chemotactic aggregation. Proc Nat Acad Sci USA 100: 13259-13263.
9. Agladze K, Wang X, Romeo T (2005) Spatial periodicity of *Escherichia coli* K-12 biofilm microstructure initiates during a reversible, polar attachment phase of development and requires the polysaccharide adhesin PGA. J Bacteriol 187: 8237-8246.
10. Romling U (2005) Characterization of the rdar morphotype, a multicellular behaviour in Enterobacteriaceae. Cell Mol Life Sci: CMLS 62: 1234-1246.
11. Monteiro C, Saxena I, Wang X, Kader A, Bokranz W, et al. (2009) Characterization of cellulose production in *Escherichia coli* Nissle 1917 and its biological consequences. Environ Microbiol 11: 1105-1116.
