# Supplementary material for: A Phyletically Rare Gene Promotes the Niche-specific Fitness of an E. coli Pathogen during Bacteremia
Source: PLoS Pathog. 2013 Feb 14;9(2):e1003175. doi: 10.1371/journal.ppat.1003175 (PMC3573123; doi:10.1371/journal.ppat.1003175)
Supplement: Text S2 — Supporting methods used in Text S1, Figure S5 and S6. (DOCX) [file ppat.1003175.s016.docx]

Supplemental Text S2: Supporting Methods

Swarm and swim motility

Swarm plates were prepared as described [1], with brief modifications using plates composed of 0.5% Eiken agar (gift from Dr. David Blair, University of Utah), 1% tryptone (Fischer), 0.5% NaCl, 0.5% yeast extract (Sigma-Aldrich), and 0.5% glucose. 5 μl of bacterial cultures, grown shaking overnight at 37°C, were inoculated onto the surface of solidified plates. Swarming was allowed to proceed for ~16 h at 37°C in a humidified incubator. Swim plates were prepared by adding agar (Sigma-Aldrich, A1296) to LB broth or defined M9 medium to a final concentration of 0.2%. The agar was melted in a microwave, taking care to not allow excessive evaporation. Agar was allowed to solidify at room temperature prior to subsurface inoculation with 1 μl of bacterial cultures that had been grown overnight at 37°C. Swimming was allowed to proceed for up to 48 h at 37°C. Results were recorded using an Epson Perfection V500 photo scanner (EPSON). Inset images in Figure 5C were captured using an SZX10 stereomicroscope, DP72 camera, and cellSens Dimension software (Olympus).

Congo red and Fluorescent Brightener 28 staining

To assess curli fiber and cellulose production, bacteria were plated on 1.2% LB- or M9 medium-agar supplemented with 0.001% Congo red dye or 50 μg/ml Fluorescent Brightener 28 (Sigma-Aldrich) incubated at 37°C. After 48 h, colonies present on the Congo red plates were imaged using an SZX10 stereomicroscope, DP72 camera, and cellSens Dimension software (Olympus). Alternatively, colonies grown overnight on

plates containing Fluorescent Brightener 28 were imaged using an iPAD2 (Apple) and an ultraviolet light box.

Preparation of bone marrow derived macrophages

Bone marrow derived macrophages (BMDM) were isolated from 6-7 week old C57BL/6 male mice from Jackson Laboratories. Mice were sacrificed by cervical dislocation while under anesthesia. Hind limbs were isolated, the muscle cut away, and bones cut to provide access to marrow. A 20-gauge needle was inserted into the marrow cavity and flushed with complete RPMI1640 media (Sigma-Aldrich) containing 10% horse serum (Gibco), L929-cleared media supernatant, 200 μM L-glutamine (Sigma Aldrich), Penicillin, Streptomycin, Gentamicin, and 2-mercaptoethanol. Isolated cells were allowed to differentiate on 100 mm^2 petri dishes and incubated for 6 days at 37^oC + 5% CO_2 in a humidified incubator with media changes on days 3 and 5 post-harvesting. For experimentation, cells were recovered from dishes by incubation with an enzyme-free cell dissociation buffer in phosphate buffered saline (PBS) solution (Gibco), and approximately 5×10^5 BMDM were plated per well in 24-well plates.

Cell association assays. Bacteria were added to BMDM monolayers at a multiplicity of infection of 10 and incubated for 1 h at 37^oC in the presence of 5% CO_2. Total numbers of surviving bacteria were determined by adding Triton X-100, at a final concentration of 0.3%, directly to each well in order to lyse the BMDMs. Lysates were then serially diluted and plated on LB agar plates to enumerate bacteria. Alternatively, levels of BMDM-associated bacteria were assessed by first washing the BMDM

monolayers with PBS (containing Ca^{2+} and Mg^{2+}), prior to lysis and plating. Data presented in Figure S6 are the pooled results from 3 independent experiments performed in triplicate. A student's *t* test was used to determined statistical significance in GraphPad Prism 5.

Reference

1. Kurihara S, Suzuki H, Oshida M, Benno Y (2011) A novel putrescine importer required for type 1 pili-driven surface motility induced by extracellular putrescine in *Escherichia coli* K-12. J Biol Chem 286: 10185-10192.
